# Development and validation of a cuproptosis-associated prognostic model for diffuse large B-cell lymphoma

**DOI:** 10.3389/fonc.2022.1020566

**Published:** 2023-01-12

**Authors:** Bingxin Zhang, Tianyu Zhang, Ziwei Zheng, Zhili Lin, Quanqiang Wang, Dong Zheng, Zixing Chen, Yongyong Ma

**Affiliations:** ^1^ Department of Hematology, The First Affiliated Hospital of Wenzhou Medical University, Wenzhou, Zhejiang, China; ^2^ Department of Hepatobiliary Surgery, The Second Affiliated Hospital and Yuying Children’s Hospital of Wenzhou Medical University, Wenzhou, Zhejiang, China

**Keywords:** diffuse large B-cell lymphoma, cuproptosis, subtypes, prognostic gene signature, overall survival, tumor microenvironment

## Abstract

Diffuse large B-cell lymphoma (DLBCL) is a highly heterogeneous disease. Therefore, more reliable biomarkers are required to better predict the prognosis of DLBCL. Cuproptosis is a novel identified form of programmed cell death (PCD) that is different from oxidative stress-related cell death (e.g., apoptosis, ferroptosis, and necroptosis) by Tsvetkov and colleagues in a recent study released in Science. Cuproptosis is copper-dependent PCD that is closely tied to mitochondrial metabolism. However, the prognostic value of cuproptosis-related genes (CRGs) in DLBCL remains to be further elucidated. In the present study, we systematically evaluated the molecular changes of CRGs in DLBCL and found them to be associated with prognosis. Subsequently, based on the expression profiles of CRGs, we characterized the heterogeneity of DLBCL by identifying two distinct subtypes using consensus clustering. Two isoforms exhibited different survival, biological functions, chemotherapeutic drug sensitivity, and immune microenvironment. After identifying differentially expressed genes (DEGs) between CRG clusters, we built a prognostic model with the Least absolute shrinkage and selection operator (LASSO) Cox regression analysis and validated its prognostic value by Cox regression analysis, Kaplan-Meier curves, and receiver operating characteristic (ROC) curves. In addition, the risk score can predict clinical characteristics, levels of immune cell infiltration, and prognosis. Furthermore, a nomogram incorporating clinical features and risk score was generated to optimize risk stratification and quantify risk assessment. Compared to the International Prognostic Index (IPI), the nomogram has demonstrated more accuracy in survival prediction. Furthermore, we validated the prognostic gene expression levels through external experiments. In conclusion, cuproptosis-related gene signature can serve as a potential prognostic predictor in DLBCL patients and may provide new insights into cancer therapeutic targets.

## 1 Introduction

Diffuse large B-cell lymphoma (DLBCL) is the most common non-Hodgkin’s lymphoma (NHL) in adults and represents a highly heterogeneous group of tumors in terms of morphology, phenotype, molecular features, clinical course, and response to therapy (1, 2). Based on gene expression profile, DLBCL can be classified from the cell of origin (COO) into at least two subtypes, germinal center B-cell-like (GCB) and activated B-cell-like (ABC), while 10-20% of cases remain unclassified (3). R-CHOP (rituximab, cyclophosphamide, doxorubicin, vincristine, and prednisone) is currently the first-line treatment for patients with DLBCL and contributes to a significant improvement in prognosis (4, 5). However, many patients of DLBCL do not respond to treatment and usually have a poor prognosis, especially in patients with the ABC subtype (6, 7). Tumor heterogeneity and the inevitable acquisition of drug resistance make DLBCL still incurable. Therefore, there is an urgent need to identify novel and reliable biomarkers that can aid in clinical risk stratification and the guidance of precision therapy.

Copper is an enzyme cofactor involved in a variety of biological functions in humans and other mammals, including cellular respiration, regulation of energy production and other redox reactions, neurotransmitter biosynthesis, and connective tissue formation (8). Moreover, copper is a key regulator of cellular signal transduction pathways, regulating or triggering multiple biological pathways in response to external stimuli (9). Therefore, the maintenance of copper homeostasis plays a crucial role in the biological activities of the organism. Many associations have been observed between cancer and copper. Several studies have reported elevated copper levels in serum and tumor tissue of patients with various malignancies, including lymphomas, compared to normal tissue (10–16). Copper accumulation has been associated with the promotion of proliferation and growth, angiogenesis, metastasis, and drug resistance (17–22). In lymphoma, serum copper level is an independent prognostic factor, closely related to tumor activity (23–25). Copper compounds are considered to be effective inducers of apoptosis in lymphoma cells (26–28). In addition, targeting mitochondria with the copper chelator drug ATN-224 may serve as an important therapeutic strategy for apoptosis-resistant DLBCL (29). A completely new form of cell death has recently been proposed that differs from all other known programmed cell death mechanisms, including apoptosis, ferroptosis, pyroptosis, and necroptosis (30). Characterized by protein lipoylation in the tricarboxylic acid (TCA) cycle, it causes acute proteotoxic stress through lipid-acylated protein aggregation and subsequent loss of iron-sulfur cluster protein, ultimately leading to cell death. Additionally, they identified seven genes (*FDX1*, *LIAS*, *LIPT1*, *DLD*, *DLAT*, *PDHA1*, and *PDHB*) that sensitized the cells to cuproptosis by genome-wide CRISPR-Cas9 loss-of-function screen, while three genes (*MTF1*, *GLS*, and *CDKN2A*) with resistance to cuproptosis. Copper importer *(SLC31A1*) and copper exporter (*ATP7B*) have also been found to promote and inhibit cuproptosis, respectively, by regulating intracellular copper concentrations (30). As we know, cuproptosis mainly targets mitochondrial respiration and the TCA cycle. In another study, the consistent cluster classification scheme identified three isoforms of DLBCL by molecular analysis, the BCR/proliferative cluster (BCR-DLBCL), OxPhos cluster (OxPhos-DLBCL), and host response cluster. Among them, the OxPhos-DLBCL was significantly enriched in genes regulating oxidative phosphorylation (OxPhos), mitochondrial membrane potential, and electron transport chain (31). A subsequent study unearthed that the OxPhos-DLBCL was insensitive to conventional drugs targeting the BCR signaling axis. With enhanced mitochondrial energy transduction, the OxPhos cluster exhibited an increased admixture of nutrient carbon in the TCA cycle (32). Furthermore, another study used immunohistochemical markers of glycolysis and mitochondrial OxPhos metabolism to explore the metabolic phenotype of human DLBCL tumors. Compared to non-tumor lymphoid tissue, the OxPhos phenotype was highly expressed in tumor lymphocytes in DLBCL samples, while stromal cells strongly expressed the glycolytic phenotype. They hypothesized that tumor cells meet their own TCA cycle substrate requirements by mediating stromal cell metabolic reorganization (33). Not surprisingly, the oxidative phosphorylation inhibitor Gboxin analog was found to have a strong proliferation inhibitory and cell cycle blocking effect on DLBCL with specific selectivity for it (34). Several recent studies have revealed the potential role of CRGs in the prognosis of cancers, such as kidney cancer (35–42), hepatocellular carcinoma (43–47), lung cancer (48–53), head and neck squamous cell carcinoma (53–60), glioma (61–66), breast cancer (67–70), endometrial carcinoma (71, 72), melanoma (73–75), pancreatic cancer (76, 77), colorectal cancer (78–81) and so on. However, no reports describe any effects of the cuproptosis regulatory mechanism on DLBCL.

This study divided 400 DLBCL samples into two subtypes based on the 12 cuproptosis-related genes (CRGs) mentioned above. The differences in survival, drug sensitivity, and immune cell infiltration between subtypes were also integrated. Subsequently, we constructed a prognostic model to stratify patients at risk. Furthermore, we developed a nomogram integrating clinical features and risk scores to quantify risk assessment and predict the overall survival (OS) of DLBCL patients. The results showed that the nomogram was an effective prognostic indicator. Finally, we performed an experimental verification on our clinical samples.

## 2 Materials and methods

### 2.1 Data acquisition

Gene expression and the relevant prognostic and clinicopathological data of DLBCL were downloaded from the public database Gene Expression Omnibus (GEO) (https://www.ncbi.nlm.nih.gov/geo/) and The Cancer Genome Atlas (TCGA) (https://portal.gdc.cancer.gov/). Microarray expression profiles of DLBCL patients were obtained from GSE10846, GSE31312, and GSE87371 datasets using Affymetrix Human Genome U133 Plus 2.0 platform. Transcriptional data for 48 DLBCL samples from TCGA were retrieved from UCSC Xena (https://xenabrowser.net/datapages/). All the microarray data included were normalized and log2 transformed. Probe IDs were mapped to gene symbols according to the corresponding annotation files, expression measurements for all probes associated with the same gene were averaged and the maximum value was finally taken. The pathology image data (formalin-fixed paraffin-embedded (FFPE) slide) were downloaded from the Genomic Data Commons (GDC; https://portal.gdc.cancer.gov/). After excluding samples with missing survival information or survival time of less than one month, the GSE10846, GSE31312, and GSE87371 included 400, 466, and 216 tumor specimens of DLBCL respectively. GSE10846 was used as the training dataset for constructing the subtype and prognostic model, while GSE31312 and TCGA-DLBCL were the validation sets for subtype identification and GSE87371 was the validation for the prognostic model. 12 CRGs (*FDX1*, *LIAS*, *LIPT1*, *DLD*, *DLAT*, *PDHA1*, *PDHB*, *MTF1*, *GLS*, *CDKN2A*, *SLC31A1*, and *ATP7B*) were obtained from the article by Tsvetkov et al. (30).

### 2.2 Clinical samples

The FFPE lymphoma tissue samples were collected from 7 patients with incipient untreated DLBCL in the First Affiliated Hospital of Wenzhou Medical University, and normal lymphoid tissues in the control group were taken from a healthy volunteer. The histological diagnosis was established according to the World Health Organization (WHO) classification (82). The study was approved by the Review Board of the First Affiliated Hospital of Wenzhou Medical University with informed consent obtained from all subjects in accordance with the Declaration of Helsinki. The clinical data of the patients are listed in **Supplementary Table 1**.

### 2.3 Gene interaction network and the effects of genetic alterations

The correlation network of 12 CRGs was derived from the “corrr” R package. To determine the somatic mutations of 12 CRGs, the single nucleotide variant (SNV) data of these 12 CRGs in all cancers, as well as the copy number variation (CNV) data in DLBCL, were mined in Gene Set Cancer Analysis (GSCA) (http://bioinfo.life.hust.edu.cn/GSCA/) (83). We also used GSCA to analyze the relationships between survival and gene expression, CNV, gene methylation, and the relationship between expression and pathway activity in DLBCL.

### 2.4 Consensus clustering analysis of CRGs

Consistent unsupervised clustering analysis was performed using the R package “ConsensusClusterPlus” (84) to classify patients based on CRG expression. Consensus clustering is based on resampling to verify the rationality of clustering, whose main purpose is to assess the stability of the clustering. The maximum number of classifications (maxK) was set to 6. The K-Means clustering algorithm was chosen and euclidean calculated the distances. 80% of the samples were resampled 1000 times by this procedure to ensure the stability and reproducibility of the classification. The optimal number of clusters k was determined by combining the graphs of each clustering result and the proportion of ambiguous clustering (PAC) method (85). GSE31312 and TCGA-DLBCL were also used for unsupervised clustering analysis to verify the accuracy of clustering. Principal component analysis (PCA) was generated by “scatterplot3d” packages to further determine the validity of the clustering. Furthermore, the differences in survival among different subtypes were assessed using Kaplan-Meier curves derived from the “survival” and “survminer” R packages. Heatmap created by the “pheatmap” software package displayed the clinical characteristics and survival differences of the different clusters.

### 2.5 Evaluation of tumor microenvironment and biological function in the cuproptosis subtypes

The infiltration fractions of 22 human immune cell subsets of every DLBCL sample were calculated by the CIBERSORT algorithm (86). Furthermore, we also used the single-sample gene set enrichment analysis (ssGSEA) algorithm (87) to validate the difference in immune cell infiltration between the subtypes in TCGA-DLBCL. Additionally, through TCGA Pathology Slides, we were able to confirm the above analysis. To investigate the differences of CRGs subtypes in biological processes, gene set variation analysis (GSVA) was performed with the Kyoto Encyclopedia of Genes and Genomes (KEGG) gene set (c2. cp. kegg. v7.2) obtained from Gene Set Enrichment Analysis (GSEA) database (http://www.gsea-msigdb.org/gsea/msigdb). Furthermore, we used the R software package “pRRophetic” (88) to evaluate the chemotherapeutic sensitivity between different subgroups.

### 2.6 Construction and validation of the prognostic signature based on the DEGs between the CRG clusters

Identification of DEGs between different subtypes using the R package “limma” (|logFC|>2, adjusted *P <*0.01) (89). Then prognosis-related DEGs were obtained by Cox regression analysis (P<0.001). Based on prognostic DEGs associated with cuproptosis, the Least absolute shrinkage and selection operator (LASSO) Cox regression analysis was used to minimize the risk of overfitting with the “glmnet” R package (90, 91). After 1000-fold cross-validation of the maximum likelihood estimate of penalty, a cuproptosis-related prognostic model was finally constructed. According to the median risk score, patients in the training and validation datasets were divided into low-risk and high-risk groups respectively, then subjected to Kaplan-Meier survival analysis. The prognostic value of the model was confirmed by univariate and multivariate Cox regression. Further validation of the model was performed with the gene expression data in lymphoma from the Cancer Cell Line Encyclopedia database (CCLE, https://portals.broadinstitute.org/ccle). Time-dependent receiver operating characteristic curve (time-ROC curve) analysis was conducted using the “timeROC” R package (92) to obtain the area under the curve (AUC) value and evaluate the predictive power of the signature. The “ggrisk” package integrated the ranking dot map, scatter map, and heatmap to show the difference in survival and gene expression between high- and low-risk groups.

### 2.7 Comprehensive analysis of CRGs-related prognostic model

The co-expression matrix of CRGs and genes in the prognostic model was established using the “ggcorrplot” package in R. For the DEGs between risk groups identified by the “limma” package, we conducted Gene Ontology (GO) enrichment analysis using the “clusterProfiler” package (93, 94). Spearman correlation analysis was used to test the correlation between tumor microenvironment (TME) and risk score.

### 2.8 Construction and evaluation of a combined nomogram

A predictive nomogram integrating the clinical characteristics and risk score was developed using the “rms” package according to the outcome of the independent prognosis analysis. Calibration plots of the nomogram were used to measure the consistency of predicted survival events and actual observed results at 1-, 2- and 3 years. Time-ROC curves for 1-, 2- and 3-year survival were performed for the assessment of accuracy in DLBCL prognosis.

### 2.9 RNA extraction and reverse transcription

RNA was extracted from the FFPE samples using the Paraffin-Embedded Tissue RNA Extraction Kit (AIDISHENG, Yancheng, China) according to the manufacturer’s instructions. Reverse transcription was performed with the cDNA synthesis kit (Vazyme, Nanjing, China) to generate cDNAs.

### 2.10 Quantitative real-time PCR

Taq Pro Universal SYBR qPCR Master Mix (Vazyme, Nanjing, China) was then used for quantitative PCR. β-ACTIN was used as an internal control, and each sample was repeated in triplicate. The relative fold-change in expression with respect to a control group was calculated by the 2-ΔΔCt method. The PCR cycle conditions were 95 °C for 30 s, followed by 40 cycles of 95 °C (10 s) and 60 °C (30 s). Three biological replicates were performed. The PCR primers used were as follows:

TUBB4A forward primer (FP): 5′‐GAGTTCCCAGACCGCATCA‐3′;
TUBB4A reverse primer (RP): 5′‐CGGAAACAGATGTCGTAGAGTG‐3′;
SLC38A5 FP: 5′‐AACAGCAATGGAGAGTGAAGC‐3′;
SLC38A5 RP: 5′‐ACCTCAGGGTGGCAGACAA‐3′;
TEX9 FP: 5′‐TTTATGAGACAGCAGCGAACA‐3′;
TEX9 RP: 5′‐GAACCTCTGTGGCACTTTGAC‐3′;
S100B FP: 5′‐GGAAGGGAGGGAGACAAGCA‐3′;
S100B RP: 5′‐CTGGAAGTCACATTCGCCGT‐3′;
ACTIN FP: 5′‐TCAAGATCATTGCTCCTCCTGAG‐3′;
ACTIN RP: 5′‐ACATCTGCTGGAAGGTGGACA‐3′;

### 2.11 Statistical analyses

All statistical analyses were performed with R version 4.1.1, GraphPad Prism 9.0.0, and SPSS software version 26.0. Student’s t-test or one-way analysis of variance was used to analyze differences between groups in variables with a normal distribution. Wilcoxon test was used to analyze the differences between groups of skewed distribution variables. And we used the Kaplan-Meier method for survival analysis and the log-rank test to analyze the differences in overall survival. *P* < 0.05 was considered statistically significant.

## 3 Results

### 3.1 Genetic alterations and interactions of CRGs in DLBCL

**Figure 1:**
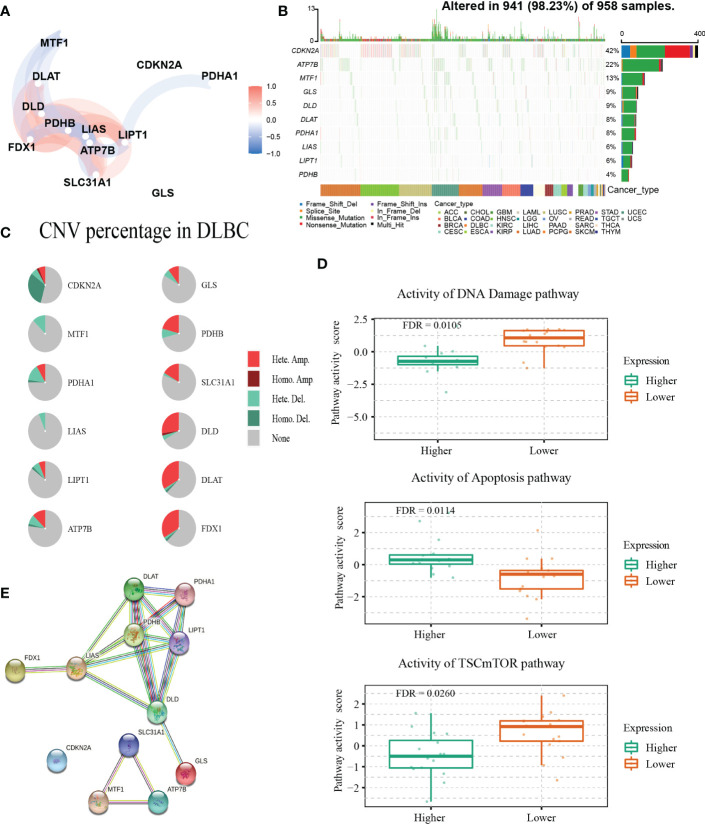
Genetic alterations and interactions of CRGs in DLBCL. **(A)** The correlation network of 12 CRGs. The correlation coefficients are represented by different colors. **(B)** The tumor mutation burden frequency of CRGs in pan-cancer analysis. **(C)** The CNV percentage of 12 CRGs in DLBCL. **(D)** The associations between expression of *SLC31A1* and activity of DNA damage and apoptosis pathway and the relationship between *CDKN2A* expression and mTOR pathway activity in DLBCL. **(E)** The PPI network encoded by CRGs in DLBCL. CNV, copy number variation; PPI, protein-protein interaction.

The detailed clinical characteristics of patients from the three GEO datasets are summarized in **Table 1**. The interaction of these 12 genes was shown in **Figure 1A**. Among the 12 CRGs, 10 of them were mutated in 98.23% (941/958) of tumor samples in pan-cancer analysis with the TCGA database (**Figure 1B**). The missense mutation was the most common mutation variant. *CDKN2A* was the most frequent mutated CRG (42%), followed by *ATP7B*, *MTF1*, *GLS*, and *DLD* (22%, 13%, 9% and 9% respectively). Next, we investigated somatic copy number changes in these CRGs of DLBCL and found widespread copy number alterations in all 12 CRGs (**Figure 1C**). Among them, the copy number variations (CNVs) of *FDX1*, *DLAT*, *DLD*, *SLC31A1*, *PDHB*, *GLS*, and *ATP7B* were mainly amplifications, while the CNVs of *CDKN2A*, *MTF1*, *PDHA1*, *LIAS*, and *LIPT1* were mainly deletions (**Figure 1C**). Furthermore, we explored the relationships between CNV, gene expression, gene methylation, and survival in the TCGA DLBCL dataset. The difference in survival between CNV and wide type was shown in **Supplementary Figure 1A**. The CNVs of *MTF1*, *GLS*, *LIPT1*, and *LIAS* were closely related to the survival of DLBCL patients (*P*<0.05). Meanwhile, the results showed that the expression of *ATP7B*, *GLS*, *MTF1*, and *LIPT1* were negatively correlated with the overall survival (OS) of DLBCL (*P*<0.05), and the first three genes were previously identified as negative regulators of cuproptosis (30). In addition to these 4 genes, the other 8 CRGs were positively correlated with OS (*P*<0.05) (**Supplementary Figure 1B**). We then explored the correlation between the expression of 12 genes and pathway activity. *SLC31A1* may have a potential activating effect on apoptosis (FDR=0.0114) and a possible inhibiting effect on DNA damage (FDR=0.0105) response (**Figure 1D**). *CDKN2A* may potentially inhibit the TSC/mTOR pathway (FDR=0.0260) (**Figure 1D**). As for methylation, hypermethylation of *SLC31A1*, *DLAT*, *DLD*, *ATP7B*, *GLS*, and *FDX1* was associated with shorter disease-specific survival (DSS) (*P*<0.05) (**Supplementary Figure 1C**). In addition, we explored the relationship between the proteins encoded by CRGs based on the Search Tool for the Retrieval of Interacting Genes (STRING) network (95) (**Figure 1E**).

**Table 1:** The clinical characteristics of the training and validation cohorts.

Characteristics	Training cohort	Validation cohort	Validation cohort
	GSE10846	GSE31312	GSE87371
	*n*=400	*n*=466	*n*=216
Gender			
Female	167(41.75%)	196(42.06%)	101(46.76%)
Male	216(54.00%)	270(57.94%)	115(53.24%)
Unknown	17(4.25%)	–	–
Age			
≤60 years	185(46.25%)	200(42.92%)	113(52.31%)
>60 years	215(53.75%)	266(57.08%)	103(47.69%)
Stage			
I- II	186(46.50%)	–	71(32.87%)
III-IV	208(52.00%)	–	145(67.13%)
Unknown	6(1.50%)	–	–
COO			
GCB	181(45.25%)	–	82(37.96%)
Non-GCB	219(54.75%)	–	134(62.04%)
ECOG PS			
0-1	291(72.75%)	372(79.83%)	–
2-4	86(21.50%)	94(20.17%)	–
Unknown	23(5.75%)	–	–
LDH			
Normal	167(41.75%)	148(31.76%)	–
>ULN	175(43.75%)	275(59.01%)	–
Unknown	58(14.50%)	43(9.23%)	–
ES			
<2	342(85.50%)	364(78.11%)	–
≥2	29(7.25%)	102(21.89%)	–
Unknown	29(7.25%)	–	–
Survival status			
OS years (median)	2.45	2.95	3.06
Censored(%)	151(37.75)	167(35.84)	45(20.83)

COO, cell of origin; GCB, germinal center B-cell-like subtype; ECOG PS, The Eastern Cooperative Oncology Group performance score; LDH, lactate dehydrogenase; ULN, the upper limit of normal; ES, extranodal sites; OS, overall survival.

### 3.2 Identification and assessment of the CRG subtypes

**Figure 2:**
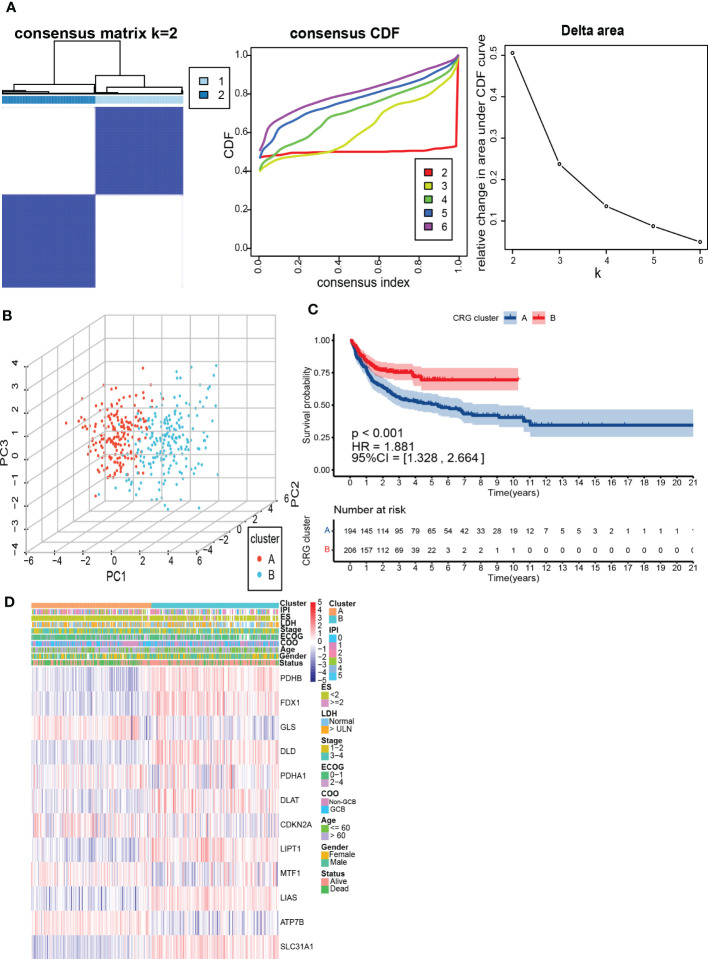
Identification and assessment of the CRG subtypes. **(A)** The consensus matrix, consensus cumulative distribution function (CDF), and delta area by cluster analysis based on CRGs. Two clusters (k=2) would be best. **(B)** Principal component analysis of two CRG clusters. **(C)** Kaplan-Meier survival curves of two CRG clusters (p < 0.001). **(D)** Heatmap of clinical features and CRGs expressions between subtypes. LDH, lactate dehydrogenases; ES, extranodal sites; ECOG, The Eastern Cooperative Oncology Group performance score; COO, cell of origin; IPI, International Prognostic Index.

To further understand the expression characteristics of CRGs in DLBCL, we used a consistent clustering algorithm to classify patients with DLBCL according to the expression profiles of 12 CRGs (**Figure 2A**). Our results indicated that k=2 appeared to be the best choice for dividing the entire cohort into A subtype (*n*=194) and B subtype (*n*=206) (**Figure 2A**). PCA revealed significant differences in the cuproptosis transcription profiles between the two subtypes (**Figure 2B**). Additionally, we used GSE31312 and TCGA-DLBCL to verify the repeatability of the clustering. Unsupervised clustering of this cohort also clearly identified 2 distinct subtypes (**Supplementary Figure 2**). The Kaplan-Meier curve showed that patients with subtype A had worse survival compared to those with subtype B (*P*<0.001; HR=1.881 [1.328, 2.664], *P*<0.001) (**Figure 2C**). Heatmap revealed differences in CRGs expression between subtypes as well as clinical features (**Figure 2D**). *GLS*, *MTF1*, and *ATP7B* were highly expressed in subtype A and were previously found to inhibit cuproptosis (30). In contrast, *PDHB*, *FDX1*, *DLD*, *DLAT*, *LIPT1*, *LIAS*, and *SLC31A1* were highly expressed in subtype B.

### 3.3 Characteristics of TME cell infiltration and biological function in the cuproptosis subtypes

**Figure 3:**
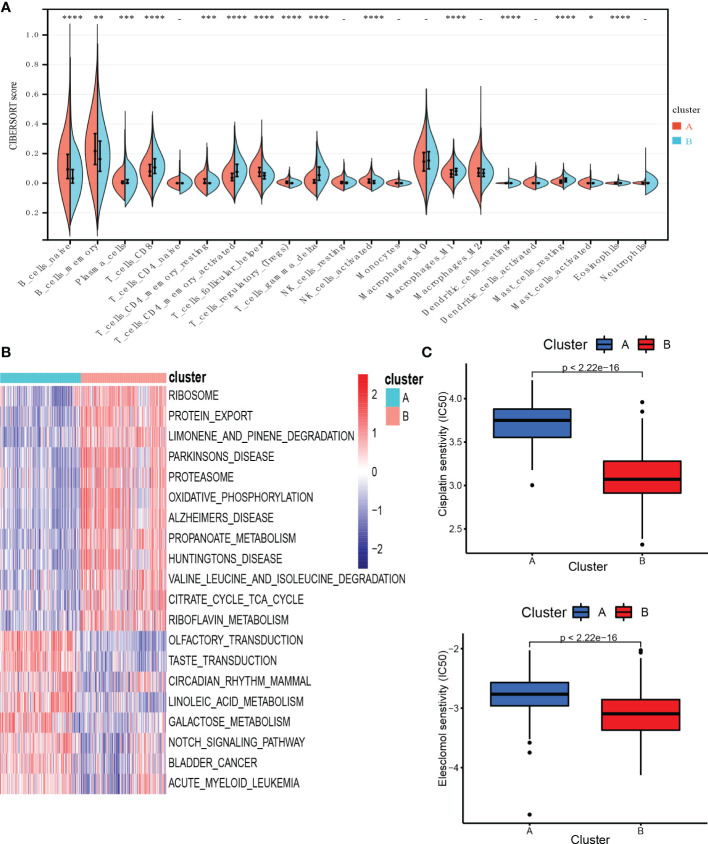
Characteristics of TME and biological function in the cuproptosis subtypes. **(A)** The abundance of different infiltrating immune cells in the two clusters. **(B)** GSVA of biological pathways between two distinct subtypes. **(C)** Prediction of drug responsiveness in the subtypes. * *P* < 0.05, ** *P* < 0.01, *** *P* < 0.001, **** *P* < 0.0001, *-* P > 0.05. TME, tumor microenvironment; IC50, half maximal inhibitory concentration.

**Figure 4:**
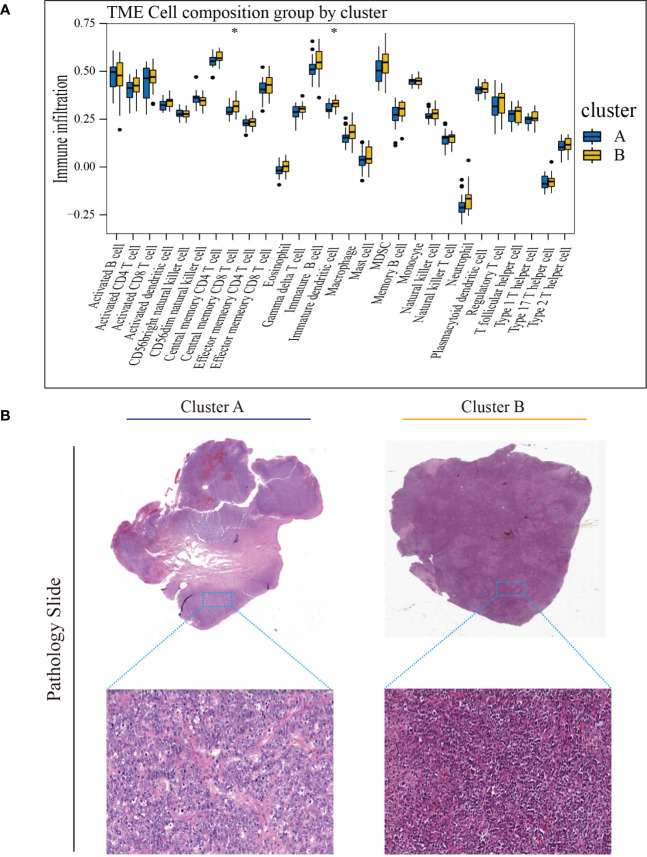
Characteristics of TME in the cuproptosis subtypes of TCGA-DLBCL. **(A)** The abundance of different infiltrating immune cells in the two clusters. **(B)** Representative images of pathological H&E staining of two cuproptosis subtypes. * *P* < 0.05; ns, not significant. TME, tumor microenvironment.

To investigate TME differences in CRGs-related subtypes, the enrichment fraction of 22 kinds of immune cells in both two clusters was evaluated using the CIBERSORT algorithm (86). Significant differences in the infiltration of most immune cells between the two subtypes were observed (**Figure 3A**). The infiltration levels of naive B cells, memory B cells, resting CD4^+^ memory T cells, regulatory T cells (Tregs), follicular helper T cells, and activated natural killer (NK) cells were higher in subtype A, while CD8^+^ T cells, activated CD4^+^ memory T cells, gamma delta T cells, M1 macrophages, plasma cells had significantly lower infiltration in subtype A compared to those in subtype B. In addition, we also carried out the ssGSEA algorithm in TCGA-DLBCL to further validate the differences in TME cell infiltration between the cuproptosis subtypes (**Figure 4A**). In conclusion, a higher level of immune infiltration was observed in subtype B. TCGA Pathology Slides further confirmed the difference (**Figure 4B**) (*P*-value < 0.05 was considered statistically significant).

To better understand the survival differences between the two subtypes, GSVA enrichment analysis of the KEGG pathway was conducted on these two clusters to assess the functional and biological differences. The results showed that the subtype A was mainly enriched in cell signal transduction pathways and immune-related pathways, such as the Notch signaling pathway, MAPK signaling pathway, VEGF signaling pathway, ERBB signaling pathway, primary immunodeficiency, and Fc epsilon RI signaling pathway. B subtype was mainly enriched in the p53 signaling pathway and metabolism-related pathways, including the metabolism of sugar, protein, fat, and nucleotide (**Figure 3B**).

We then evaluated the therapeutic responsiveness of chemotherapeutic agents between the two subgroups, subtype A exhibited a resistance tendency to bleomycin, cisplatin, doxorubicin, etoposide, vincristine, vinorelbine and elesclomol, a kind of copper ionophore, while B was more resistant to paclitaxel and methotrexate (**Figure 3C**; **Supplementary Figure 3**) (*P*-value < 0.05 was considered statistically significant).

### 3.4 Construction and validation of the prognostic signature based on the DEGs between the CRG clusters

**Figure 5:**
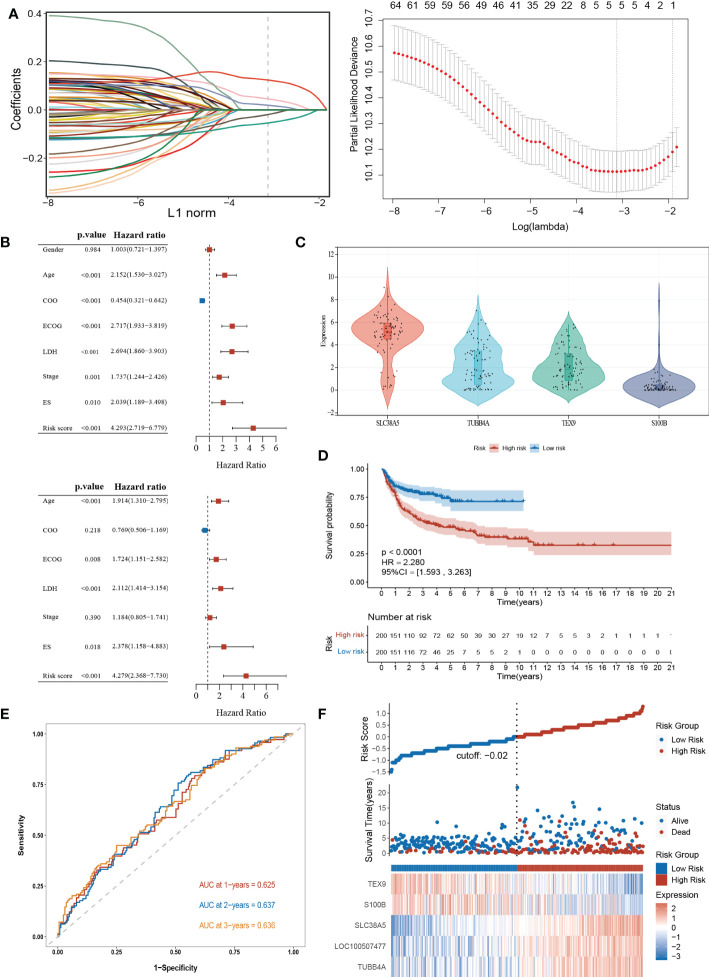
Construction and validation of the prognostic signature according to cuproptosis-related DEGs. **(A)** Construction of the prognostic model using LASSO Cox regression analysis. **(B)** Univariate and multivariate Cox regression analysis of clinical features and risk score in the training dataset. **(C)** Expression of the 4 genes in the CCLE database. **(D)** Kaplan-Meier curve in the high- and low-risk group. **(E)** Sensitivity and specificity of the risk score model assessed by time-dependent ROC analysis. **(F)** Ranked dot and scatter plots showing the risk score distribution and patients’ survival status. LASSO, the Least absolute shrinkage and selection operator; ROC, receiver operating characteristic. LDH, lactate dehydrogenases; ES, extranodal sites; ECOG, The Eastern Cooperative Oncology Group performance score.

To explore the potential biological functions of the cuproptosis subgroups in DLBCL, we identified the DEGs between the two clusters with the “limma” R package, and 180 genes were obtained (|logFC|>2, adjusted P <0.01). Univariable Cox regression analysis was then performed to acquire 66 DEGs associated with prognosis (p< 0.001). Finally, the candidate genes were incorporated into the construction of the prognostic model using LASSO Cox regression analysis, and a signature of 5 genes (*S100B*, *TUBB4A*, *SLC38A5*, *LOC100507477*, and *TEX9*) was discovered. The optimal weighting coefficient for each gene was determined by the regularization parameter lambda using the min standard (**Figure 5A**).

The risk model was constructed as follows: risk score = (0.1239 × expression of *SLC38A5*) + (0.0194 × expression of *TUBB4A*) + (0.0458 × expression of *LOC100507477)* – (0.0192 × expression of *S100B)* – (0.0541× expression of *TEX9*). According to the median risk score of the corresponding datasets, the patients with DLBCL were divided into high-scoring and low-scoring groups respectively in training and validation datasets.

Multivariate Cox regression analysis showed that the risk score was an independent prognostic factor for DLBCL (**Figure 5B**). Next, we performed the external validation on the expression of these four genes in lymphoma using the online database CCLE (no data was available for *LOC100507477*), and the expression levels of *S100B* and *TEX9* were relatively low, consistent with the above formula (**Figure 5C**). The Kaplan-Meier survival curve showed a significant difference between the groups. The high-risk group had a poor prognosis in the GSE10846 (*P*<0.0001; HR=2.280 [1.593, 3.263], *P*<0.001) (**Figure 5D**). In the GSE87371, the ability of the model to predict patient prognosis was further verified (*P*=0.040; HR=1.829 [1.019, 3.282], *P*=0.043) (**Supplementary Figure 4A**). Additionally, the 1-year, 2-year, and 3-year survival predicted by the risk score exhibited AUC values of 0.625, 0.637, and 0.636, respectively (**Figure 5E**). AUCs for the validation dataset were shown in **Supplementary Figure 4B**. The ranked dot demonstrated that the prognostic model can well distinguish high-risk groups from others. The scatter plot further revealed the worse survival outcome in the high-scoring group compared to the low-scoring group (**Figure 5F**). The same was true in the validation queue (**Supplementary Figure 4C**).

### 3.5 Comprehensive analysis of cuproptosis-related prognostic model

To further analyze the differences between high- and low-risk groups, we investigated the relationship between different clinical characteristics and risk scores. The risk scores were higher in those LDH above normal levels and non-GCB subgroups (*P*<0.05) (**Supplementary Figure 5A**). And these factors were also identified as risk factors for poor prognosis of DLBCL in previous studies (96). A high score was also closely associated with subtype A (*P*<0.05), consistent with the previous results of poor prognosis for subtype A (**Supplementary Figure 5A**).

**Figure 6:**
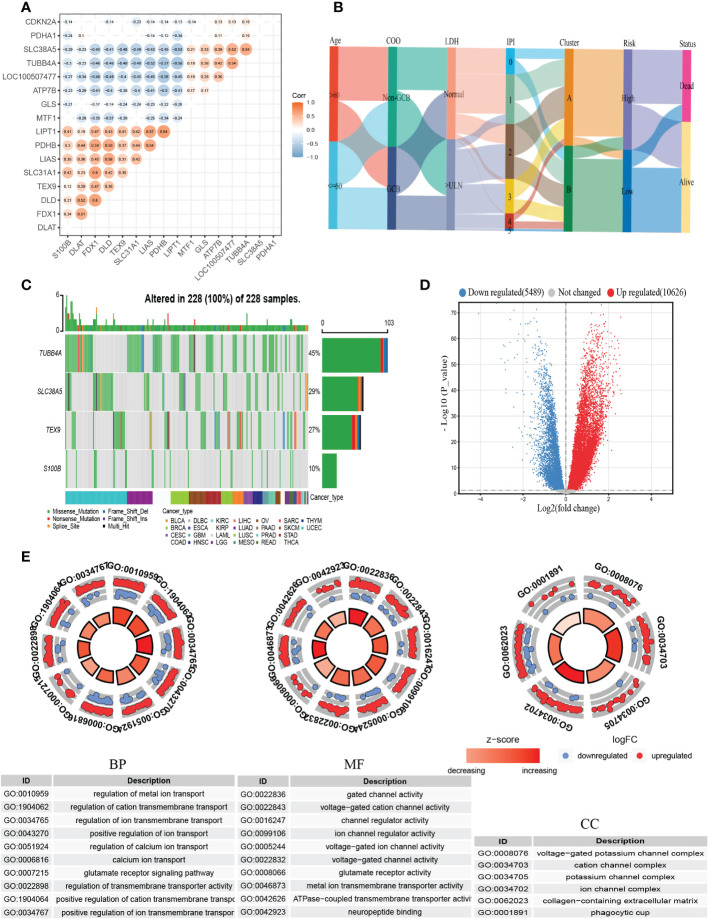
Comprehensive analysis of the cuproptosis-related prognostic model. **(A)** Correlations between CRGs and the prognostic model. The results of *P*>0.05 were not shown. **(B)** Alluvial diagram showing clinical characteristics distribution and survival differences of CRG clusters and risk groups. **(C)** The tumor mutation burden frequency of genes in the prognostic model in the pan-cancer analysis. **(D)** The DEGs between high- and low-risk groups. **(E)** GO enrichment analyses of DEGs. COO, cell of origin; LDH, lactate dehydrogenases; IPI, International Prognostic Index; DEGs, differentially expressed genes; GO, Gene Ontology; BP, biological process; CC, cellular component; MF, molecular function.

Then we constructed the co-expression matrix of CRGs and genes in the prognostic model to further explore the association between cuproptosis and the model. The results revealed widespread correlations in these genes (**Figure 6A**). For instance, *LIPT1* and *PDHB* (*r*=0.64, *P*<0.05) were positively correlated, while *LIPT1* was negatively correlated with *TUBB4A* (*r*=−0.58, *P*<0.05). Alluvial diagram is plotted for a better display of clinical characteristics and survival differences between CRG clusters and risk groups (**Figure 6B**).

To better understand the difference in survival in the prognostic signature, the analysis of the molecular alterations in five genes of the prognostic model was performed. The summary report showed that four of them were mutated at a high frequency in tumor specimens (**Figure 6C**). Mutations were present in all 228 samples (100%) (**Figure 6C**). Among them, *TUBB4A* had the highest mutation frequency (45%), followed by *SLC38A5* (29%). The proportion of the CNV for each gene was summarized in **Supplementary Figure 5B**.

To further explore the biological differences between different risk groups, we investigated the DEGs between the two groups, which generated 2624 genes (|logFC|>1, adjusted *P <*0.01) (**Figure 6D**). GO enrichment analysis indicated that the functions of the DEGs were predominantly related to ion transport, such as regulation of metal ion transport and regulation of transmembrane transporter activity (**Figure 6E**).

The association between CRGs and the abundance of immune cells had been previously demonstrated, and here we also investigated the correlation between DEGs-related risk core and immune cell infiltration. As shown in the correlation scatter plot, Tregs, follicular helper T cells, activated and resting NK cells, naive B cells, memory B cells, and activated dendritic cells were positively correlated with the risk score. However, a negative correlation was found between the risk score and gamma delta T cells, activated memory CD4^+^ T cells, CD8^+^ T cells, M1 macrophages, resting mast cells, and resting dendritic cells (**Supplementary Figure 5C**).

### 3.6 Construction and evaluation of combined nomogram

**Figure 7:**
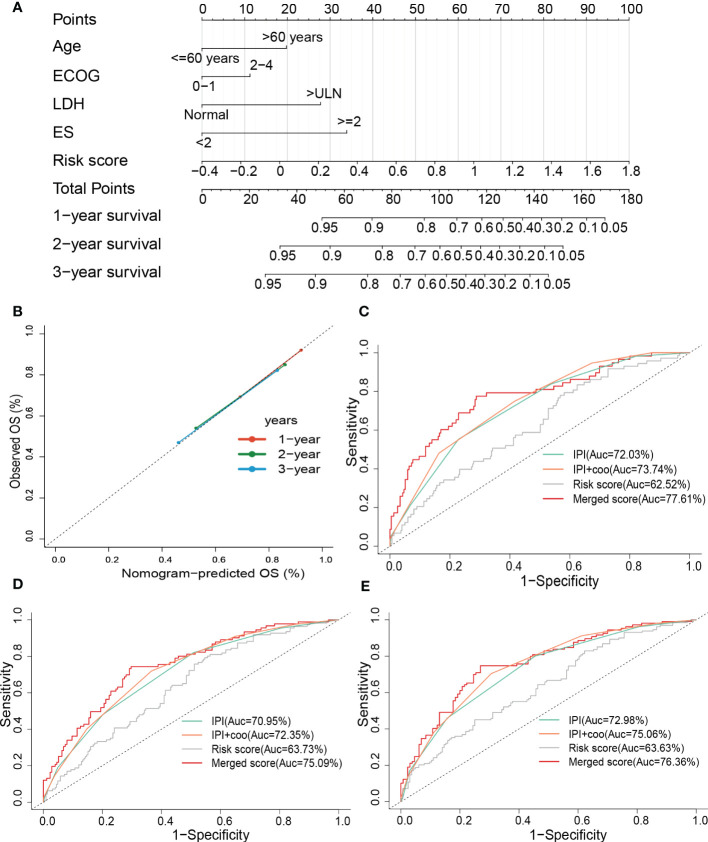
Constructing and evaluating the combined nomogram. **(A)** The nomograms with age, LDH, ES, ECOG, and risk score. **(B)** Calibration curves of the nomogram for predicting 1-, 2-, and 3-year survival. ROC curves of the nomogram, risk score, COO+IPI, and IPI score for **(C)** 1-year and **(D)** 2-year, and **(E)** 3-year survival prediction. LDH, lactate dehydrogenases; ES, extranodal sites; ECOG, The Eastern Cooperative Oncology Group performance score; ROC, receiver operating characteristic; COO, cell of origin; IPI, International Prognostic Index.

To improve the predictive accuracy as well as the clinical utility of the prognostic model, we constructed a nomogram based on multivariate Cox regression analysis, and the nomogram incorporated age, the Eastern Cooperative Oncology Group (ECOG), lactate dehydrogenase (LDH), extranodal sites (ES), and risk sore (**Figure 7A**). A C-index of 0.735 indicated that the nomogram had a good predictive value. The calibration plot for survival probability exhibited a satisfactory consensus between the prediction and observation (**Figure 7B**). The 1-, 2- and 3-year AUC of the nomogram were 77.61%, 75.09%, and 76.36%, respectively, higher than the AUC of the cell of origin (COO) plus/or International Prognostic Index (IPI) alone (**Figures 7C–E**). IPI is the current prognostic benchmark for DLBCL.

### 3.7 External experimental validation of prognostic genes

**Figure 8:**
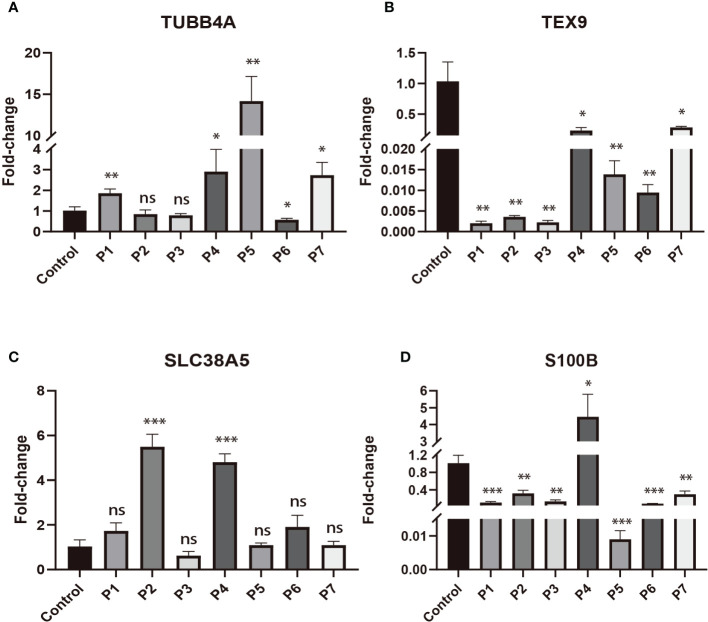
The expressions of four signature genes were validated by quantitative real-time PCR (qRT-PCR). *P< 0.05; **P< 0.01; ***P< 0.001; ns, no significance. **(A)** The expression of TUBB4A in DLBCL patients. **(B)** The expression of TEX9 in DLBCL patients. **(C)** The expression of SLC38A5 in DLBCL patients. **(D)** The expression of S100B in DLBCL patients.

To validate the expression changes of these prognostic signature genes in patients with DLBCL, we collected 7 clinical samples of DLBCL and detected the mRNA expression of these four genes (*TUBB4A*, *SLC38A5*, *S100B*, and *TEX9*) using qRT-PCR. The trend of gene expression in the qRT-PCR analysis was basically consistent with our prognostic model. Compared with the normal lymphoid tissue in the control sample, the expression of risk genes (*TUBB4A* and *SLC38A5*) was up-regulated, while the expression of protective genes (*S100B* and *TEX9*) was down-regulated in most DLBCL samples (*P* < 0.05) (**Figures 8A–D**).

## 4 Discussion

Copper is a trace metal essential to life. The amount of copper in the organism is strictly controlled. Due to the close relationship between copper and the occurrence and development of cancer, copper ionophores (disulfiram/DSF, dithiocarbamates, elesclomol, etc.) and copper chelators (trientine, tetrathiomolybdate, etc.) have been applied in anticancer treatment (97, 98). Cu-DSF has strong cytotoxicity to leukemic stem cell-like cells in a dose-dependent manner, whereas it does not affect normal hematopoietic progenitor cells (99). This may be related to the ability of cancer cells to accumulate copper, but also to their oxidative sensitivity. Elesclomol has been included in the clinical trial for acute myeloid leukemia (100). Copper chelating agents, known to have anti-angiogenic and anti-tumor activity, have been demonstrated the rate-limiting effects on tumor growth (101–104). Choline tetrathiomolybdate (ATN-224) was found to induce mitochondrial dysfunction and caspase-independent cell death in DLBCL (29). Recently, a novel mode of cell death that relies on the TCA cycle and mitochondrial oxidative respiration has been proposed, called cuproptosis (30). TCA cycle and OxPhos are also known to play important roles in DLBCL. B-cell lymphoma uses glucose and glutamine to fuel the TCA cycle for producing energy and metabolic precursors to support cell growth and proliferation (32, 105). Glucose-independent glutamine metabolism promotes the proliferation and survival of human lymphoma cells through the TCA cycle (105). Impaired TCA cycling has been reported to induce autophagy (106), which acts as a tumor suppressor in DLBCL (107). Currently, efforts to capture the molecular heterogeneity of DLBCL depend on gene expression profiles. In another transcriptomic approach, consensus cluster classification identified three subgroups, the BCR/proliferative cluster (BCR-DLBCL), showing upregulation of genes encoding B-cell receptor (BCR) signaling components, OxPhos cluster (OxPhos-DLBCL), which was significantly enriched in mitochondrial OxPhos-related genes, host response tumors were characterized by active host inflammatory infiltration (31). The OxPhos subset was characterized by non-functional BCR signaling and increased mitochondrial metabolism. Compared with BCR-DLBCL, OxPhos-DLBCL showed enhanced mitochondrial energy transduction, greater incorporation of nutrient-derived carbon into the TCA cycle, and consequent activation of antioxidant defense mechanisms (108). The OxPhos molecular marker provided these subpopulations with alternative survival benefits independent of the BCR network (32). In a study of the metabolic phenotype of DLBCL, neoplastic lymphocytes in DLBCL samples expressed a significant OxPhos phenotype, whereas stromal cells strongly exhibited a glycolytic phenotype, compared with non-tumor lymphoid tissues from control samples. This suggests that tumor lymphocytes in DLBCL undergo a significant degree of mitochondrial oxidative metabolism rather than aerobic glycolysis. They then hypothesized that DLBCL’s multi-compartment metabolism is a result of adapting to its high metabolic demands. Neoplastic cells metabolically reorganize the surrounding stroma to undergo aerobic glycolysis, providing them with substrates for the TCA cycle (33). As an OxPhos inhibitor, the Gboxin analog 5d has specific selectivity for DLBCL. Given its strong proliferation inhibition and cell cycle-blocking effects on DLBCL, 5d is considered a candidate agent for DLBCL alternative drug development (34). In conclusion, the TCA cycle and mitochondrial oxidative respiration are closely associated with DLBCL. However, the role of cuproptosis in DLBCL has not been explored.

Identifying tumor subgroups with different pathogenesis and possible therapeutic targets to characterize the genetic heterogeneity of DLBCL will facilitate a deeper understanding and precise treatment of the disease. In this study, we identified two isoforms by consensus clustering based on the expression profiles of 12 CRGs. Meanwhile, we explored the CNV, gene expression, and methylation of 12 CRGs in the TCGA DLBCL dataset. Due to the small number of samples in the dataset (*n*=48), we performed a pan-cancer analysis of the SNVs in 12 CRGs. The results showed that 10 of 12 genes were mutated in 98.23% (941/958) of tumor samples, of which *CDKN2A* was the most (42%). In addition, CNVs were present in all 12 CRGs. Among them, the most frequent were *CDKN2A* (predominantly deletion) and *FDX1* (predominantly amplification). *FDX1* is known to regulate protein lipoylation and is a key regulator in the process of cuproptosis (30). Furthermore, the CNVs of *MTF1*, *GLS*, *LIPT1*, and *LIAS* were closely related to the survival time of DLBCL patients. We then found that the high expressions of 3 genes identified as cuproptosis-negative regulators (*MTF1*, *GLS*, and *ATP7B*) (30) were associated with shorter OS. In addition, in the study of the correlation between gene expression and pathway activity, *SLC31A1* was found to have a potential activating effect on the apoptosis pathway and a potentially inhibiting effect on DNA damage. And *CDKN2A* exhibited potential inhibition of the TSC/mTOR pathway. All of these pathways are known to play a critical role in tumor growth and progression. *SLC31A1* and *ATP7B* are copper importer (109, 110) and copper efflux transporter, respectively (30). *SLC31A1* has been reported to be an important pathway for platinum drug import into cells (111). In addition, a lower expression level of *SLC31A1* is usually associated with increased cisplatin resistance in tumors (112). Conversely, *ATP7B* is involved in the efflux and sequestration of cisplatin, thereby increasing the resistance of tumor cells to platinum treatment (113, 114). *CDKN2A*, an important tumor suppressor gene, which is frequently mutated or absent in a variety of tumors, is capable of inducing cell cycle arrest in G1 and G2 phases (115). *CDKN2A* deletion is the most common gene copy number abnormality in DLBCL, which is associated with poor prognosis (116–118). Metal-responsive transcription factor-1 (*MTF1*) is a candidate susceptibility gene for lymphoma. Since the products of targets such as metallothionein can suppress cellular stress generated by ionizing radiation (119). Glutaminase (*GLS*) is a mitochondrial enzyme that catalyzes the conversion of glutamine to glutamate (120). Highly expressed in cancers including lymphoma, blocking its enzymatic activity or gene knockout has been shown to have antitumor activity (121, 122). Under the stress of PD-1-expressing γδ T cells, *GLS* confers immunosuppressive properties to ABC-DLBCL cells by enhancing mitochondrial bioenergetics and consequent STAT3 activation and PD-L1 expression in ABC-DLBCL cells (123). These analyses above have demonstrated the potential role of cuproptosis in the prognosis of DLBCL.

In the subsequent comparative analysis of the two subtypes, we found that subtype A had poorer survival than B, and the four cuproptosis-negative regulatory genes (*CDKN2A*, *MTF1*, *GLS*, and *ATP7B*) were more highly expressed in subtype A. The difference in the expression of CRGs between the two subtypes may be a potential reason for their distinct prognosis. Next, potential biological differences between subtypes were explored by GSVA. The results showed that subtype A was mainly enriched in pathways closely related to tumor growth and development, such as the Notch signaling pathway, MAPK signaling pathway, VEGF signaling pathway, and ERBB signaling pathway. For instance, Notch2 is a key membrane receptor for B-cell function and plays a critical role in the pathogenesis of lymphoma (124). The main enriched pathways of subtype B include the p53 signaling pathway and metabolism-related pathways, such as oxidative phosphorylation and the TCA cycle. Subtype B may be closely related to OxPhos-DLBCL identified by Monti et al. (31).

Given the increasing importance of TME in cancer treatment and prognosis, immune cell infiltration between the two subtypes was assessed by the CIBERSORT algorithm. Tregs and resting CD4^+^ memory T cells were more infiltrated in subtype A, whereas CD8^+^ T cells, gamma delta T cells, and CD4^+^ memory-activated T cells were more infiltrated in subtype B. Similarly, the immune infiltration ssGSEA scores in TCGA-DLBCL and the pathological slide data showed a higher immune infiltration in cluster B. In the subsequent drug sensitivity prediction analysis, subgroup A exhibited resistance to doxorubicin, bleomycin, etoposide, elesclomol, and cisplatin. Resistance to cisplatin may be related to higher expression of *ATP7B* and lower expression of *SLC31A1* in subgroup A. Differences in immune status and treatment responsiveness between subtypes may contribute to the differences in survival outcomes.

Based on DEGs between cuproptosis-related subpopulations, we constructed and validated a prognostic model integrating five genes (*S100B*, *TEX9*, *TUBB4A*, *SLC38A5*, *LOC100507477*) using LASSO regression analysis. The model was identified as an independent prognostic factor in Cox regression analysis. Different clinical characteristics and prognoses were demonstrated between high- and low-risk groups. The prognosis of the high-risk group was worse compared to the low-risk group. Subtype A, LDH>ULN, and non-GCB subpopulations tended to have a higher risk score.

Furthermore, four of the five genes in the prognostic model had SNVs in all 228 samples (100%) of the pan-cancer analysis, with the most variant being *TUBB4A* (45%). All four genes had copy number abnormalities in patients with DLBCL. The human microtubulin β-IVa class (*TUBB4A*), which pertains to the β-microtubulin family, has little or no expression in most normal tissues but is highly expressed in a variety of human cancer cell lines (125). *TUBB4A* deletion has been reported to reduce prostate tumor growth and metastasis by inhibiting the activation of NF-κB, cell cycle protein D1, and c-MYC signaling (126). It is also involved in the resistance of multiple cancer cells to chemotherapy and radiotherapy (127–129). Likewise, *TEX9* is a testis-expressed protein that belongs to cancer/testis antigen (CTA) and is normally expressed only in the testis, except in early-developing embryos and the placenta. *TEX9* expression can be induced in tumor cells when cancer occurs. *TEX9* has been shown the promotion of proliferation and migration and has an inhibitory effect on the apoptosis of esophageal squamous carcinoma cells (130). *S100* calcium-binding protein B (*S100B*) is a Ca^+2^/Zn^+2^-*p53* binding protein that blocks phosphorylation and acetylation sites on *p53* important for transcriptional activation (131). In addition, it appears as a key signaling molecule in many physiological and pathological processes, including inflammation, apoptosis, and cell growth (132). High expression of *S100B* in antigen-presenting cells correlates with a good prognosis (133). *SLC38A5* is a sodium-coupled transporter upregulated in multiple cancers, mediating the influx of glutamine, serine, glycine, and methionine into cancer cells. It responds to the metabolic reprogramming of cancer cells to meet the expansive demands of tumor growth and proliferation (134). Furthermore, the gene co-expression matrix revealed the close association of 12 CRGs with genes in the prognostic model. However, the relationship between these genes in model and DLBCL still needs to be further investigated.

According to GO enrichment analysis, DEGs between high- and low-risk groups were closely associated with the metal ion transport-related pathways. This suggests that there may be differences in copper transport capacity between the high and low-risk groups, which may be one of the potential reasons for the different prognoses in the two groups. Since serum copper levels have previously been shown to be positively correlated with disease status in non-Hodgkin lymphoma, with significantly higher levels in active or relapsed patients than in patients in remission (16, 23–25).

Correlation analysis of risk score and immune cell infiltration indicated that the risk score was positively correlated with the infiltration of Tregs, but negatively correlated with the abundance of gamma delta T cells, CD8^+^ T cells, and activated CD4^+^ memory T cells. This may have led to the difference in survival between the high- and low-risk groups. Ultimately, a nomogram integrating clinical features and risk scores was constructed. IPI is the main clinical tool used to predict the prognosis of patients with aggressive NHL and is the current prognostic benchmark for DLBCL (2, 96). Compared with IPI, the nomogram demonstrated higher accuracy and discrimination in predicting survival.

To further verify the expression level of signature genes in DLBCL, we performed qRT-PCR on our clinical samples to quantify the mRNA expression of these four genes (*TUBB4A*, *SLC38A5*, *S100B*, and *TEX9*). qRT-PCR analysis showed that the expression of risk genes (*TUBB4A* and *SLC38A5)* was up-regulated, while the expression of protective genes (*S100B* and *TEX9*) was down-regulated in DLBCL. This was generally consistent with our prognostic model.

Our study has several limitations. Firstly, our clustering typing strategy and prognostic signature need to be further validated for their robustness and clinical utility in a larger sample. Secondly, since the establishment and validation of the prognostic model were primarily based on public databases, further validations are required through cell experiments and larger clinical samples. In addition, the specific mechanism of CRG in DLBCL and the underlying mechanism between CRGs and tumor immunity in DLBCL are currently unclear and require further study.

In conclusion, we performed systematic analyses on the molecular alterations of CRGs in DLBCL, and our study suggests that these genes may play a key role in the prognosis of DLBCL. The two subtypes identified based on the CRGs expression signature were significantly different in biological function, immune cell infiltration, treatment responsiveness, and clinical prognosis. In addition, the prognostic model constructed from CRG performed well in predicting the survival of DLBCL patients and was significantly correlated with the level of immune infiltration. Furthermore, we built a nomogram combining clinical features and risk scores that improved the predictive power of DLBCL. Finally, we carried out an external experiment to verify the level of prognostic gene expression. Our work provides new directions for prognostic prediction and potential therapeutic targets in DLBCL and may provide the basis for more in-depth studies in the future.

## Data availability statement

The original contributions presented in the study are included in the article/**Supplementary Material**. Further inquiries can be directed to the corresponding author.

## Author contributions

BZ conceived the study design, completed data analysis and interpretation, and manuscript writing. QW assisted in the completion of experiments. TZ, ZZ, ZL, DZ, and ZC provided suggestions for the data analysis. YM provided critical revisions to the paper. All authors contributed to the article and approved the submitted version.

## Supplementary material

Supplementary Figure 1
**(A)** Correlation analysis of CNV and survival in DLBCL. **(B)** The relationships between expression of CRGs and survival in DLBCL. **(C)** Correlation analysis of gene methylation of CRGs and survival in DLBCL. The bubble color from blue to red represents the hazard ratio from low to high, and bubble size is positively correlated with the Cox P value significance. The black outline border indicates Cox P value ≤ 0.05.

Supplementary Figure 2 Validation of CRG clusters. **(A, C)** The consensus matrix of the consensus clustering. **(B, D)** The sample clustering consistency diagram. A and B were for GSE31312. C and D were for TCGA-DLBCL. Two clusters (k=2) would be best.

Supplementary Figure 3 Prediction of drug responsiveness in the distinct subtypes. IC50, half maximal inhibitory concentration.

Supplementary Figure 4 Validation of the prognostic signature in GSE87371. **(A)** Kaplan-Meier curve in the high- and low-risk group. **(B)** Sensitivity and specificity of the risk score model assessed by time-depend ROC analysis. **(C)** Ranked dot and scatter plots showing the risk score distribution and patients’ survival status.

Supplementary Figure 5
**(A)** Differences in risk scores among groups with distinct clinical characteristics. **(B)** The CNV percentage of genes in the model of DLBCL. **(C)** Correlation between risk score and immune infiltration in DLBCL. COO, cell of origin; LDH, lactate dehydrogenases; CNV, copy number variation.

## References

[1] Chan WC Armitage JO Gascoyne R Connors J Close P Jacobs P. A clinical evaluation of the international lymphoma study group classification of non-hodgkin's lymphoma. the non-hodgkin's lymphoma classification project. Blood (1997) 89(11):3909–18. doi: 10.1182/blood.V89.11.3909 9166827

[2] Sehn LH Salles G. Diffuse large b-cell lymphoma. N Engl J Med (2021) 384(9):842–58. doi: 10.1056/NEJMra2027612 PMC8377611 33657296

[3] Alizadeh AA Eisen MB Davis RE Ma C Lossos IS Rosenwald A. Distinct types of diffuse large b-cell lymphoma identified by gene expression profiling. Nature (2000) 403(6769):503–11. doi: 10.1038/35000501 10676951

[4] Coiffier B Lepage E Briere J Herbrecht R Tilly H Bouabdallah R. Chop chemotherapy plus rituximab compared with chop alone in elderly patients with diffuse large-b-cell lymphoma. N Engl J Med (2002) 346(4):235–42. doi: 10.1056/NEJMoa011795 11807147

[5] Feugier P Van Hoof A Sebban C Solal-Celigny P Bouabdallah R Ferme C. Long-term results of the r-chop study in the treatment of elderly patients with diffuse large b-cell lymphoma: a study by the groupe d'etude des lymphomes de l'adulte. J Clin Oncol (2005) 23(18):4117–26. doi: 10.1200/JCO.2005.09.131 15867204

[6] Karube K Enjuanes A Dlouhy I Jares P Martin-Garcia D Nadeu F. Integrating genomic alterations in diffuse large b-cell lymphoma identifies new relevant pathways and potential therapeutic targets. Leukemia (2018) 32(3):675–84. doi: 10.1038/leu.2017.251 PMC5843901 28804123

[7] Lenz G Wright G Dave SS Xiao W Powell J Zhao H. Stromal gene signatures in large-b-cell lymphomas. N Engl J Med (2008) 359(22):2313–23. doi: 10.1056/NEJMoa0802885 PMC9103713 19038878

[8] Wachnik A. The physiological role of copper and the problems of copper nutritional deficiency. Nahrung (1988) 32(8):755–65. doi: 10.1002/food.19880320811 3068548

[9] Grubman A White AR. Copper as a key regulator of cell signalling pathways. Expert Rev Mol Med (2014) 16:e11. doi: 10.1017/erm.2014.11 24849048

[10] Yaman M Kaya G Yekeler H. Distribution of trace metal concentrations in paired cancerous and non-cancerous human stomach tissues. World J Gastroenterol (2007) 13(4):612–8. doi: 10.3748/wjg.v13.i4.612 PMC4065986 17278230

[11] Yaman M Kaya G Simsek M. Comparison of trace element concentrations in cancerous and noncancerous human endometrial and ovary tissues. Int J Gynecol Cancer (2007) 17(1):220–8. doi: 10.1111/j.1525-1438.2006.00742.x 17291257

[12] Mao S Huang S. Zinc and copper levels in bladder cancer: A systematic review and meta-analysis. Biol Trace Elem Res (2013) 153(1-3):5–10. doi: 10.1007/s12011-013-9682-z 23640281

[13] Ressnerova A Raudenska M Holubova M Svobodova M Polanska H Babula P. Zinc and copper homeostasis in head and neck cancer: Review and meta-analysis. Curr Med Chem (2016) 23(13):1304–30. doi: 10.2174/0929867323666160405111543 27048341

[14] Lener MR Scott RJ Wiechowska-Kozlowska A Serrano-Fernandez P Baszuk P Jaworska-Bieniek K. Serum concentrations of selenium and copper in patients diagnosed with pancreatic cancer. Cancer Res Treat (2016) 48(3):1056–64. doi: 10.4143/crt.2015.282 PMC4946347 26727715

[15] Shen F Cai WS Li JL Feng Z Cao J Xu B. The association between serum levels of selenium, copper, and magnesium with thyroid cancer: A meta-analysis. Biol Trace Elem Res (2015) 167(2):225–35. doi: 10.1007/s12011-015-0304-9 25820485

[16] Gozdasoglu S Cavdar AO Arcasoy A Akar N. Serum copper and zinc levels and copper/zinc ratio in pediatric non-hodgkin's lymphoma. Acta Haematol (1982) 67(1):67–70. doi: 10.1159/000207027 6800204

[17] Ishida S Andreux P Poitry-Yamate C Auwerx J Hanahan D. Bioavailable copper modulates oxidative phosphorylation and growth of tumors. Proc Natl Acad Sci U.S.A. (2013) 110(48):19507–12. doi: 10.1073/pnas.1318431110 PMC3845132 24218578

[18] Vella V Malaguarnera R Lappano R Maggiolini M Belfiore A. Recent views of heavy metals as possible risk factors and potential preventive and therapeutic agents in prostate cancer. Mol Cell Endocrinol (2017) 457:57–72. doi: 10.1016/j.mce.2016.10.020 27773847

[19] Tisato F Marzano C Porchia M Pellei M Santini C. Copper in diseases and treatments, and copper-based anticancer strategies. Med Res Rev (2010) 30(4):708–49. doi: 10.1002/med.20174 19626597

[20] De Luca A Barile A Arciello M Rossi L. Copper homeostasis as target of both consolidated and innovative strategies of anti-tumor therapy. J Trace Elem Med Biol (2019) 55:204–13. doi: 10.1016/j.jtemb.2019.06.008 31345360

[21] Shanbhag V Jasmer-McDonald K Zhu S Martin AL Gudekar N Khan A. Atp7a delivers copper to the lysyl oxidase family of enzymes and promotes tumorigenesis and metastasis. Proc Natl Acad Sci U.S.A. (2019) 116(14):6836–41. doi: 10.1073/pnas.1817473116 PMC6452744 30890638

[22] Arnesano F Natile G. Interference between copper transport systems and platinum drugs. Semin Cancer Biol (2021) 76:173–88. doi: 10.1016/j.semcancer.2021.05.023 34058339

[23] Cohen Y Epelbaum R Haim N McShan D Zinder O. The value of serum copper levels in non-hodgkin's lymphoma. Cancer (1984) 53(2):296–300. doi: 10.1002/1097-0142(19840115)53:2<296::aid-cncr2820530219>3.0.co;2-u 6690011

[24] Hisamitsu S Shibuya H Hoshina M Horiuchi J. Prognostic factors in head and neck non-hodgkin's lymphoma with special reference to serum lactic dehydrogenase and serum copper. Acta Oncol (1990) 29(7):879–83. doi: 10.3109/02841869009096383 2261202

[25] Shah-Reddy I Khilanani P Bishop CR. Serum copper levels in non-hodgkin's lymphoma. Cancer (1980) 45(8):2156–9. doi: 10.1002/1097-0142(19800415)45:8<2156::aid-cncr2820450824>3.0.co;2-c 6989485

[26] Spengler G Kincses A Racz B Varga B Watanabe G Saijo R. Benzoxazole-based Zn(Ii) and Cu(Ii) complexes overcome multidrug-resistance in cancer. Anticancer Res (2018) 38(11):6181–7. doi: 10.21873/anticanres.12971 30396935

[27] Ng CH Kong SM Tiong YL Maah MJ Sukram N Ahmad M. Selective anticancer copper(ii)-mixed ligand complexes: targeting of ros and proteasomes. Metallomics (2014) 6(4):892–906. doi: 10.1039/c3mt00276d 24549332

[28] Easmon J Purstinger G Heinisch G Roth T Fiebig HH Holzer W. Synthesis, cytotoxicity, and antitumor activity of copper(ii) and iron(ii) complexes of (4)n-azabicyclo[3.2.2]nonane thiosemicarbazones derived from acyl diazines. J Med Chem (2001) 44(13):2164–71. doi: 10.1021/jm000979z 11405653

[29] Lee K Hart MR Briehl MM Mazar AP Tome ME. The copper chelator atn-224 induces caspase-independent cell death in diffuse large b cell lymphoma. Int J Oncol (2014) 45(1):439–47. doi: 10.3892/ijo.2014.2396 PMC4079159 24788952

[30] Tsvetkov P Coy S Petrova B Dreishpoon M Verma A Abdusamad M. Copper induces cell death by targeting lipoylated tca cycle proteins. Science (2022) 375(6586):1254–61. doi: 10.1126/science.abf0529 PMC9273333 35298263

[31] Monti S Savage KJ Kutok JL Feuerhake F Kurtin P Mihm M. Molecular profiling of diffuse large b-cell lymphoma identifies robust subtypes including one characterized by host inflammatory response. Blood (2005) 105(5):1851–61. doi: 10.1182/blood-2004-07-2947 15550490

[32] Caro P Kishan AU Norberg E Stanley IA Chapuy B Ficarro SB. Metabolic signatures uncover distinct targets in molecular subsets of diffuse large b cell lymphoma. Cancer Cell (2012) 22(4):547–60. doi: 10.1016/j.ccr.2012.08.014 PMC3479446 23079663

[33] Gooptu M Whitaker-Menezes D Sprandio J Domingo-Vidal M Lin Z Uppal G. Mitochondrial and glycolytic metabolic compartmentalization in diffuse large b-cell lymphoma. Semin Oncol (2017) 44(3):204–17. doi: 10.1053/j.seminoncol.2017.10.002 PMC5737785 29248132

[34] Yao S Yin J Liu W Li Y Huang J Qi C. A novel gboxin analog induces oxphos inhibition and mitochondrial dysfunction-mediated apoptosis in diffuse large b-cell lymphoma. Bioorg Chem (2022) 127:106019. doi: 10.1016/j.bioorg.2022.106019 35849895

[35] Bian Z Fan R Xie L. A novel cuproptosis-related prognostic gene signature and validation of differential expression in clear cell renal cell carcinoma. Genes (Basel) (2022) 13(5):851. doi: 10.3390/genes13050851 35627236 PMC9141858

[36] Ji ZH Ren WZ Wang HQ Gao W Yuan B. Molecular subtyping based on cuproptosis-related genes and characterization of tumor microenvironment infiltration in kidney renal clear cell carcinoma. Front Oncol (2022) 12:919083. doi: 10.3389/fonc.2022.919083 35875087 PMC9299088

[37] Mei W Liu X Jia X Jin L Xin S Sun X. A cuproptosis-related gene model for predicting the prognosis of clear cell renal cell carcinoma. Front Genet (2022) 13:905518. doi: 10.3389/fgene.2022.905518 36092880 PMC9450221

[38] Li K Tan L Li Y Lyu Y Zheng X Jiang H. Cuproptosis identifies respiratory subtype of renal cancer that confers favorable prognosis. Apoptosis (2022) 27(11-12):1004–14. doi: 10.1007/s10495-022-01769-2 36103026

[39] Zhang G Chen X Fang J Tai P Chen A Cao K. Cuproptosis status affects treatment options about immunotherapy and targeted therapy for patients with kidney renal clear cell carcinoma. Front Immunol (2022) 13:954440. doi: 10.3389/fimmu.2022.954440 36059510 PMC9437301

[40] Yuan H Qin X Wang J Yang Q Fan Y Xu D. The cuproptosis-associated 13 gene signature as a robust predictor for outcome and response to immune- and targeted-therapies in clear cell renal cell carcinoma. Front Immunol (2022) 13:971142. doi: 10.3389/fimmu.2022.971142 36131921 PMC9483097

[41] Xu S Liu D Chang T Wen X Ma S Sun G. Cuproptosis-associated lncrna establishes new prognostic profile and predicts immunotherapy response in clear cell renal cell carcinoma. Front Genet (2022) 13:938259. doi: 10.3389/fgene.2022.938259 35910212 PMC9334800

[42] Huili Y Nie S Zhang L Yao A Liu J Wang Y. Cuproptosis-related lncrna: prediction of prognosis and subtype determination in clear cell renal cell carcinoma. Front Genet (2022) 13:958547. doi: 10.3389/fgene.2022.958547 36072656 PMC9441767

[43] Wang Y Zhang Y Wang L Zhang N Xu W Zhou J. Development and experimental verification of a prognosis model for cuproptosis-related subtypes in hcc. Hepatol Int (2022) 16(6):1435–47. doi: 10.1007/s12072-022-10381-0 36065073

[44] Liu Y Liu Y Ye S Feng H Ma L. Development and validation of cuproptosis-related gene signature in the prognostic prediction of liver cancer. Front Oncol (2022) 12:985484. doi: 10.3389/fonc.2022.985484 36033443 PMC9413147

[45] Zhang G Sun J Zhang X. A novel cuproptosis-related lncrna signature to predict prognosis in hepatocellular carcinoma. Sci Rep (2022) 12(1):11325. doi: 10.1038/s41598-022-15251-1 35790864 PMC9256635

[46] Zhang Z Zeng X Wu Y Liu Y Zhang X Song Z. Cuproptosis-related risk score predicts prognosis and characterizes the tumor microenvironment in hepatocellular carcinoma. Front Immunol (2022) 13:925618. doi: 10.3389/fimmu.2022.925618 35898502 PMC9311491

[47] Fu J Wang S Li Z Qin W Tong Q Liu C. Comprehensive multiomics analysis of cuproptosis-related gene characteristics in hepatocellular carcinoma. Front Genet (2022) 13:942387. doi: 10.3389/fgene.2022.942387 36147507 PMC9486098

[48] Yun Y Wang Y Yang E Jing X. Cuproptosis-related gene - slc31a1, fdx1 and atp7b polymorphisms are associated with risk of lung cancer. Pharmgenomics Pers Med (2022) 15:733–42. doi: 10.2147/PGPM.S372824 PMC9342429 35923305

[49] Wang F Lin H Su Q Li C. Cuproptosis-related lncrna predict prognosis and immune response of lung adenocarcinoma. World J Surg Oncol (2022) 20(1):275. doi: 10.1186/s12957-022-02727-7 36050740 PMC9434888

[50] Hu Q Wang R Ma H Zhang Z Xue Q. Cuproptosis predicts the risk and clinical outcomes of lung adenocarcinoma. Front Oncol (2022) 12:922332. doi: 10.3389/fonc.2022.922332 36003780 PMC9393616

[51] Zhang H Shi Y Yi Q Wang C Xia Q Zhang Y. A novel defined cuproptosis-related gene signature for predicting the prognosis of lung adenocarcinoma. Front Genet (2022) 13:975185. doi: 10.3389/fgene.2022.975185 36046242 PMC9421257

[52] Gao C Kong N Zhang F Zhou L Xu M Wu L. Development and validation of the potential biomarkers based on m6a-related lncrnas for the predictions of overall survival in the lung adenocarcinoma and differential analysis with cuproptosis. BMC Bioinf (2022) 23(1):327. doi: 10.1186/s12859-022-04869-7 PMC9358839 35941550

[53] Wang S Xing N Meng X Xiang L Zhang Y. Comprehensive bioinformatics analysis to identify a novel cuproptosis-related prognostic signature and its cerna regulatory axis and candidate traditional chinese medicine active ingredients in lung adenocarcinoma. Front Pharmacol (2022) 13:971867. doi: 10.3389/fphar.2022.971867 36110528 PMC9468865

[54] Xu M Mu J Wang J Zhou Q Wang J. Construction and validation of a cuproptosis-related lncrna signature as a novel and robust prognostic model for colon adenocarcinoma. Front Oncol (2022) 12:961213. doi: 10.3389/fonc.2022.961213 35965536 PMC9367690

[55] Zhang S Zhang L Lu H Yao Y Liu X Hou J. A cuproptosis and copper metabolism-related gene prognostic index for head and neck squamous cell carcinoma. Front Oncol (2022) 12:955336. doi: 10.3389/fonc.2022.955336 36072790 PMC9441563

[56] Tang S Zhao L Wu XB Wang Z Cai LY Pan D. Identification of a novel cuproptosis-related gene signature for prognostic implication in head and neck squamous carcinomas. Cancers (Basel) (2022) 14(16):3986. doi: 10.3390/cancers14163986 36010978 PMC9406337

[57] Li G Luo Q Wang X Zeng F Feng G Che G. Deep learning reveals cuproptosis features assist in predict prognosis and guide immunotherapy in lung adenocarcinoma. Front Endocrinol (Lausanne) (2022) 13:970269. doi: 10.3389/fendo.2022.970269 36060936 PMC9437348

[58] Ding Q Chen X Hong W Wang L Liu W Cai S. The prognostic role of cuproptosis in head and neck squamous cell carcinoma patients: a comprehensive analysis. Dis Markers (2022) 2022:9996946. doi: 10.1155/2022/9996946 36092958 PMC9463014

[59] Yang L Yu J Tao L Huang H Gao Y Yao J. Cuproptosis-related lncrnas are biomarkers of prognosis and immune microenvironment in head and neck squamous cell carcinoma. Front Genet (2022) 13:947551. doi: 10.3389/fgene.2022.947551 35938003 PMC9354258

[60] Chen Y. Identification and validation of cuproptosis-related prognostic signature and associated regulatory axis in uterine corpus endometrial carcinoma. Front Genet (2022) 13:912037. doi: 10.3389/fgene.2022.912037 35937995 PMC9353190

[61] Chen B Zhou X Yang L Zhou H Meng M Zhang L. A cuproptosis activation scoring model predicts neoplasm-immunity interactions and personalized treatments in glioma. Comput Biol Med (2022) 148:105924. doi: 10.1016/j.compbiomed.2022.105924 35964468

[62] Bao JH Lu WC Duan H Ye YQ Li JB Liao WT. Identification of a novel cuproptosis-related gene signature and integrative analyses in patients with lower-grade gliomas. Front Immunol (2022) 13:933973. doi: 10.3389/fimmu.2022.933973 36045691 PMC9420977

[63] Wang W Lu Z Wang M Liu Z Wu B Yang C. The cuproptosis-related signature associated with the tumor environment and prognosis of patients with glioma. Front Immunol (2022) 13:998236. doi: 10.3389/fimmu.2022.998236 36110851 PMC9468372

[64] Yan X Wang N Dong J Wang F Zhang J Hu X. A cuproptosis-related lncrnas signature for prognosis, chemotherapy, and immune checkpoint blockade therapy of low-grade glioma. Front Mol Biosci (2022) 9:966843. doi: 10.3389/fmolb.2022.966843 36060266 PMC9428515

[65] Ye Z Zhang S Cai J Ye L Gao L Wang Y. Development and validation of cuproptosis-associated prognostic signatures in who 2/3 glioma. Front Oncol (2022) 12:967159. doi: 10.3389/fonc.2022.967159 36059638 PMC9434124

[66] Ouyang Z Zhang H Lin W Su J Wang X. Bioinformatic profiling identifies the glutaminase to be a potential novel cuproptosis-related biomarker for glioma. Front Cell Dev Biol (2022) 10:982439. doi: 10.3389/fcell.2022.982439 36158220 PMC9500213

[67] Sha S Si L Wu X Chen Y Xiong H Xu Y. Prognostic analysis of cuproptosis-related gene in triple-negative breast cancer. Front Immunol (2022) 13:922780. doi: 10.3389/fimmu.2022.922780 35979353 PMC9376234

[68] Li X Ma Z Mei L. Cuproptosis-related gene slc31a1 is a potential predictor for diagnosis, prognosis and therapeutic response of breast cancer. Am J Cancer Res (2022) 12(8):3561–80. PMC9442001 36119835

[69] Li L Li L Sun Q. High expression of cuproptosis-related slc31a1 gene in relation to unfavorable outcome and deregulated immune cell infiltration in breast cancer: an analysis based on public databases. BMC Bioinf (2022) 23(1):350. doi: 10.1186/s12859-022-04894-6 PMC9394027 35996075

[70] Cheng T Wu Y Liu Z Yu Y Sun S Guo M. Cdkn2a-mediated molecular subtypes characterize the hallmarks of tumor microenvironment and guide precision medicine in triple-negative breast cancer. Front Immunol (2022) 13:970950. doi: 10.3389/fimmu.2022.970950 36052076 PMC9424905

[71] Shan J Geng R Zhang Y Wei J Liu J Bai J. Identification of cuproptosis-related subtypes, establishment of a prognostic model and tumor immune landscape in endometrial carcinoma. Comput Biol Med (2022) 149:105988. doi: 10.1016/j.compbiomed.2022.105988 36007289

[72] Lei L Tan L Sui L. A novel cuproptosis-related gene signature for predicting prognosis in cervical cancer. Front Genet (2022) 13:957744. doi: 10.3389/fgene.2022.957744 36092887 PMC9453033

[73] Lv H Liu X Zeng X Liu Y Zhang C Zhang Q. Comprehensive analysis of cuproptosis-related genes in immune infiltration and prognosis in melanoma. Front Pharmacol (2022) 13:930041. doi: 10.3389/fphar.2022.930041 35837286 PMC9273972

[74] Zhou Y Shu Q Fu Z Wang C Gu J Li J. A novel risk model based on cuproptosis-related lncrnas predicted prognosis and indicated immune microenvironment landscape of patients with cutaneous melanoma. Front Genet (2022) 13:959456. doi: 10.3389/fgene.2022.959456 35938036 PMC9354044

[75] Yang X Wang X Sun X Xiao M Fan L Su Y. Construction of five cuproptosis-related lncrna signature for predicting prognosis and immune activity in skin cutaneous melanoma. Front Genet (2022) 13:972899. doi: 10.3389/fgene.2022.972899 36160015 PMC9490379

[76] Huang X Zhou S Toth J Hajdu A. Cuproptosis-related gene index: a predictor for pancreatic cancer prognosis, immunotherapy efficacy, and chemosensitivity. Front Immunol (2022) 13:978865. doi: 10.3389/fimmu.2022.978865 36090999 PMC9453428

[77] Liu T Liu Q Wang Y Yang R Tian F. Cuproptosis scoring model predicts overall survival and assists in immunotherapeutic decision making in pancreatic carcinoma. Front Genet (2022) 13:938488. doi: 10.3389/fgene.2022.938488 36118866 PMC9472214

[78] Li Y Wang RY Deng YJ Wu SH Sun X Mu H. Molecular characteristics, clinical significance, and cancer immune interactions of cuproptosis and ferroptosis-associated genes in colorectal cancer. Front Oncol (2022) 12:975859. doi: 10.3389/fonc.2022.975859 36132144 PMC9483209

[79] Hou D Tan JN Zhou SN Yang X Zhang ZH Zhong GY. A novel prognostic signature based on cuproptosis-related lncrna mining in colorectal cancer. Front Genet (2022) 13:969845. doi: 10.3389/fgene.2022.969845 36105091 PMC9465626

[80] Du Y Lin Y Wang B Li Y Xu D Gan L. Cuproptosis patterns and tumor immune infiltration characterization in colorectal cancer. Front Genet (2022) 13:976007. doi: 10.3389/fgene.2022.976007 36176287 PMC9513614

[81] Wang L Cao Y Guo W Xu J. High expression of cuproptosis-related gene fdx1 in relation to good prognosis and immune cells infiltration in colon adenocarcinoma (COAD). J Cancer Res Clin Oncol [Preprint] (2022). doi: 10.1007/s00432-022-04382-7 PMC9889456 36173462

[82] Swerdlow SH Campo E Pileri SA Harris NL Stein H Siebert R. The 2016 revision of the world health organization classification of lymphoid neoplasms. Blood (2016) 127(20):2375–90. doi: 10.1182/blood-2016-01-643569 PMC4874220 26980727

[83] Liu CJ Hu FF Xia MX Han L Zhang Q Guo AY. Gscalite: A web server for gene set cancer analysis. Bioinformatics (2018) 34(21):3771–2. doi: 10.1093/bioinformatics/bty411 29790900

[84] Wilkerson MD Hayes DN. Consensusclusterplus: A class discovery tool with confidence assessments and item tracking. Bioinformatics (2010) 26(12):1572–3. doi: 10.1093/bioinformatics/btq170 PMC2881355 20427518

[85] Senbabaoglu Y Michailidis G Li JZ. Critical limitations of consensus clustering in class discovery. Sci Rep (2014) 4:6207. doi: 10.1038/srep06207 25158761 PMC4145288

[86] Sturm G Finotello F Petitprez F Zhang JD Baumbach J Fridman WH. Comprehensive evaluation of transcriptome-based cell-type quantification methods for immuno-oncology. Bioinformatics (2019) 35(14):i436–i45. doi: 10.1093/bioinformatics/btz363 PMC6612828 31510660

[87] Rooney MS Shukla SA Wu CJ Getz G Hacohen N. Molecular and genetic properties of tumors associated with local immune cytolytic activity. Cell (2015) 160(1-2):48–61. doi: 10.1016/j.cell.2014.12.033 25594174 PMC4856474

[88] Geeleher P Cox N Huang RS. Prrophetic: An r package for prediction of clinical chemotherapeutic response from tumor gene expression levels. PloS One (2014) 9(9):e107468. doi: 10.1371/journal.pone.0107468 25229481 PMC4167990

[89] Ritchie ME Phipson B Wu D Hu Y Law CW Shi W. Limma powers differential expression analyses for rna-sequencing and microarray studies. Nucleic Acids Res (2015) 43(7):e47. doi: 10.1093/nar/gkv007 25605792 PMC4402510

[90] Friedman J Hastie T Tibshirani R. Regularization paths for generalized linear models *via* coordinate descent. J Stat Softw (2010) 33(1):1–22. 20808728 PMC2929880

[91] Simon N Friedman J Hastie T Tibshirani R. Regularization paths for cox's proportional hazards model *via* coordinate descent. J Stat Softw (2011) 39(5):1–13. doi: 10.18637/jss.v039.i05 PMC4824408 27065756

[92] Blanche P Dartigues JF Jacqmin-Gadda H. Estimating and comparing time-dependent areas under receiver operating characteristic curves for censored event times with competing risks. Stat Med (2013) 32(30):5381–97. doi: 10.1002/sim.5958 24027076

[93] Yu G Wang LG Han Y He QY. Clusterprofiler: An r package for comparing biological themes among gene clusters. OMICS (2012) 16(5):284–7. doi: 10.1089/omi.2011.0118 PMC3339379 22455463

[94] Wu T Hu E Xu S Chen M Guo P Dai Z. Clusterprofiler 4.0: A universal enrichment tool for interpreting omics data. Innovation (Camb) (2021) 2(3):100141. doi: 10.1016/j.xinn.2021.100141 34557778 PMC8454663

[95] Szklarczyk D Franceschini A Kuhn M Simonovic M Roth A Minguez P. The string database in 2011: functional interaction networks of proteins, globally integrated and scored. Nucleic Acids Res (2011) 39(Database issue):D561–8. doi: 10.1093/nar/gkq973 PMC3013807 21045058

[96] International Non-Hodgkin's Lymphoma Prognostic Factors P. A predictive model for aggressive non-hodgkin's lymphoma. N Engl J Med (1993) 329(14):987–94. doi: 10.1056/NEJM199309303291402 8141877

[97] Babak MV Ahn D. Modulation of intracellular copper levels as the mechanism of action of anticancer copper complexes: clinical relevance. Biomedicines (2021) 9(8):852. doi: 10.3390/biomedicines9080852 34440056 PMC8389626

[98] Oliveri V. Selective targeting of cancer cells by copper ionophores: An overview. Front Mol Biosci (2022) 9:841814. doi: 10.3389/fmolb.2022.841814 35309510 PMC8931543

[99] Xu B Wang S Li R Chen K He L Deng M. Disulfiram/copper selectively eradicates aml leukemia stem cells in vitro and in vivo by simultaneous induction of ros-jnk and inhibition of nf-kappab and Nrf2. Cell Death Dis (2017) 8(5):e2797. doi: 10.1038/cddis.2017.176 28518151 PMC5520701

[100] Hedley D Shamas-Din A Chow S Sanfelice D Schuh AC Brandwein JM. A phase i study of elesclomol sodium in patients with acute myeloid leukemia. Leuk Lymphoma (2016) 57(10):2437–40. doi: 10.3109/10428194.2016.1138293 26732437

[101] Brem SS Zagzag D Tsanaclis AM Gately S Elkouby MP Brien SE. Inhibition of angiogenesis and tumor growth in the brain. suppression of endothelial cell turnover by penicillamine and the depletion of copper, an angiogenic cofactor. Am J Pathol (1990) 137(5):1121–42. PMC1877678 1700617

[102] Yoshii J Yoshiji H Kuriyama S Ikenaka Y Noguchi R Okuda H. The copper-chelating agent, trientine, suppresses tumor development and angiogenesis in the murine hepatocellular carcinoma cells. Int J Cancer (2001) 94(6):768–73. doi: 10.1002/ijc.1537 11745476

[103] Pan Q Kleer CG van Golen KL Irani J Bottema KM Bias C. Copper deficiency induced by tetrathiomolybdate suppresses tumor growth and angiogenesis. Cancer Res (2002) 62(17):4854–9. 12208730

[104] Cox C Teknos TN Barrios M Brewer GJ Dick RD Merajver SD. The role of copper suppression as an antiangiogenic strategy in head and neck squamous cell carcinoma. Laryngoscope (2001). doi: 10.1097/00005537-200104000-00024 11359142

[105] Le A Lane AN Hamaker M Bose S Gouw A Barbi J. Glucose-independent glutamine metabolism *via* tca cycling for proliferation and survival in b cells. Cell Metab (2012) 15(1):110–21. doi: 10.1016/j.cmet.2011.12.009 PMC3345194 22225880

[106] Martinez-Reyes I Diebold LP Kong H Schieber M Huang H Hensley CT. Tca cycle and mitochondrial membrane potential are necessary for diverse biological functions. Mol Cell (2016) 61(2):199–209. doi: 10.1016/j.molcel.2015.12.002 26725009 PMC4724312

[107] Li M Chiang YL Lyssiotis CA Teater MR Hong JY Shen H. Non-oncogene addiction to sirt3 plays a critical role in lymphomagenesis. Cancer Cell (2019) 35(6):916–31 e9. doi: 10.1016/j.ccell.2019.05.002 31185214 PMC7534582

[108] Chen L Monti S Juszczynski P Daley J Chen W Witzig TE. Syk-dependent tonic b-cell receptor signaling is a rational treatment target in diffuse large b-cell lymphoma. Blood (2008) 111(4):2230–7. doi: 10.1182/blood-2007-07-100115 PMC2234057 18006696

[109] Ishida S Lee J Thiele DJ Herskowitz I. Uptake of the anticancer drug cisplatin mediated by the copper transporter ctr1 in yeast and mammals. Proc Natl Acad Sci U.S.A. (2002) 99(22):14298–302. doi: 10.1073/pnas.162491399 PMC137878 12370430

[110] Lin X Okuda T Holzer A Howell SB. The copper transporter ctr1 regulates cisplatin uptake in saccharomyces cerevisiae. Mol Pharmacol (2002) 62(5):1154–9. doi: 10.1124/mol.62.5.1154 12391279

[111] Holzer AK Manorek GH Howell SB. Contribution of the major copper influx transporter ctr1 to the cellular accumulation of cisplatin, carboplatin, and oxaliplatin. Mol Pharmacol (2006) 70(4):1390–4. doi: 10.1124/mol.106.022624 16847145

[112] Song IS Savaraj N Siddik ZH Liu P Wei Y Wu CJ. Role of human copper transporter ctr1 in the transport of platinum-based antitumor agents in cisplatin-sensitive and cisplatin-resistant cells. Mol Cancer Ther (2004) 3(12):1543–9. 15634647

[113] Komatsu M Sumizawa T Mutoh M Chen ZS Terada K Furukawa T. Copper-transporting p-type adenosine triphosphatase (atp7b) is associated with cisplatin resistance. Cancer Res (2000) 60(5):1312–6. 10728692

[114] Samimi G Safaei R Katano K Holzer AK Rochdi M Tomioka M. Increased expression of the copper efflux transporter atp7a mediates resistance to cisplatin, carboplatin, and oxaliplatin in ovarian cancer cells. Clin Cancer Res (2004) 10(14):4661–9. doi: 10.1158/1078-0432.CCR-04-0137 15269138

[115] Liggett WH Jr. Sidransky D. Role of the P16 tumor suppressor gene in cancer. J Clin Oncol (1998) 16(3):1197–206. doi: 10.1200/JCO.1998.16.3.1197 9508208

[116] Jardin F Ruminy P Kerckaert JP Parmentier F Picquenot JM Quief S. Detection of somatic quantitative genetic alterations by multiplex polymerase chain reaction for the prediction of outcome in diffuse large b-cell lymphomas. Haematologica (2008) 93(4):543–50. doi: 10.3324/haematol.12251 18287131

[117] Jardin F Jais JP Molina TJ Parmentier F Picquenot JM Ruminy P. Diffuse large b-cell lymphomas with cdkn2a deletion have a distinct gene expression signature and a poor prognosis under r-chop treatment: a gela study. Blood (2010) 116(7):1092–104. doi: 10.1182/blood-2009-10-247122 20435884

[118] Lenz G Wright GW Emre NC Kohlhammer H Dave SS Davis RE. Molecular subtypes of diffuse large b-cell lymphoma arise by distinct genetic pathways. Proc Natl Acad Sci U.S.A. (2008) 105(36):13520–5. doi: 10.1073/pnas.0804295105 PMC2533222 18765795

[119] Tamura Y Maruyama M Mishima Y Fujisawa H Obata M Kodama Y. Predisposition to mouse thymic lymphomas in response to ionizing radiation depends on variant alleles encoding metal-responsive transcription factor-1 (Mtf-1). Oncogene (2005) 24(3):399–406. doi: 10.1038/sj.onc.1208197 15516976

[120] Wise DR DeBerardinis RJ Mancuso A Sayed N Zhang XY Pfeiffer HK. Myc regulates a transcriptional program that stimulates mitochondrial glutaminolysis and leads to glutamine addiction. Proc Natl Acad Sci U.S.A. (2008) 105(48):18782–7. doi: 10.1073/pnas.0810199105 PMC2596212 19033189

[121] Wang JB Erickson JW Fuji R Ramachandran S Gao P Dinavahi R. Targeting mitochondrial glutaminase activity inhibits oncogenic transformation. Cancer Cell (2010) 18(3):207–19. doi: 10.1016/j.ccr.2010.08.009 PMC3078749 20832749

[122] Gross MI Demo SD Dennison JB Chen L Chernov-Rogan T Goyal B. Antitumor activity of the glutaminase inhibitor cb-839 in triple-negative breast cancer. Mol Cancer Ther (2014) 13(4):890–901. doi: 10.1158/1535-7163.MCT-13-0870 24523301

[123] Xia X Zhou W Guo C Fu Z Zhu L Li P. Glutaminolysis mediated by malt1 protease activity facilitates pd-l1 expression on abc-dlbcl cells and contributes to their immune evasion. Front Oncol (2018) 8:632. doi: 10.3389/fonc.2018.00632 30619766 PMC6305595

[124] Zhang X Shi Y Weng Y Lai Q Luo T Zhao J. The truncate mutation of notch2 enhances cell proliferation through activating the nf-kappab signal pathway in the diffuse large b-cell lymphomas. PloS One (2014) 9(10):e108747. doi: 10.1371/journal.pone.0108747 25314575 PMC4196756

[125] Luduena RF. Are tubulin isotypes functionally significant. Mol Biol Cell (1993) 4(5):445–57. doi: 10.1091/mbc.4.5.445 PMC300949 8334301

[126] Gao S Wang S Zhao Z Zhang C Liu Z Ye P. Tubb4a interacts with myh9 to protect the nucleus during cell migration and promotes prostate cancer *via* Gsk3beta/Beta-catenin signalling. Nat Commun (2022) 13(1):2792. doi: 10.1038/s41467-022-30409-1 35589707 PMC9120517

[127] Atjanasuppat K Lirdprapamongkol K Jantaree P Svasti J. Non-adherent culture induces paclitaxel resistance in h460 lung cancer cells *via* erk-mediated up-regulation of betaiva-tubulin. Biochem Biophys Res Commun (2015) 466(3):493–8. doi: 10.1016/j.bbrc.2015.09.057 26375501

[128] Tamura D Arao T Nagai T Kaneda H Aomatsu K Fujita Y. Slug increases sensitivity to tubulin-binding agents *via* the downregulation of betaiii and betaiva-tubulin in lung cancer cells. Cancer Med (2013) 2(2):144–54. doi: 10.1002/cam4.68 PMC3639653 23634282

[129] Kavallaris M Kuo DY Burkhart CA Regl DL Norris MD Haber M. Taxol-resistant epithelial ovarian tumors are associated with altered expression of specific beta-tubulin isotypes. J Clin Invest (1997) 100(5):1282–93. doi: 10.1172/JCI119642 PMC508306 9276747

[130] Xu F Zhang S Liu Z Gu J Li Y Wang L. Tex9 and Eif3b functionally synergize to promote the progression of esophageal squamous cell carcinoma. BMC Cancer (2019) 19(1):875. doi: 10.1186/s12885-019-6071-9 31481019 PMC6724304

[131] van Dieck J Teufel DP Jaulent AM Fernandez-Fernandez MR Rutherford TJ Wyslouch-Cieszynska A. Posttranslational modifications affect the interaction of s100 proteins with tumor suppressor P53. J Mol Biol (2009) 394(5):922–30. doi: 10.1016/j.jmb.2009.10.002 19819244

[132] Donato R Sorci G Riuzzi F Arcuri C Bianchi R Brozzi F. S100b's double life: intracellular regulator and extracellular signal. Biochim Biophys Acta (2009) 1793(6):1008–22. doi: 10.1016/j.bbamcr.2008.11.009 19110011

[133] Chan WJ. Pathogenesis of diffuse large b cell lymphoma. Int J Hematol (2010) 92(2):219–30. doi: 10.1007/s12185-010-0602-0 20582737

[134] Sniegowski T Korac K Bhutia YD Ganapathy V. Slc6a14 and slc38a5 drive the glutaminolysis and serine-glycine-one-carbon pathways in cancer. Pharm (Basel) (2021) 14(3):216. doi: 10.3390/ph14030216 PMC8000594 33806675

