# Supplementary material for: Development and validation of a cuproptosis-associated prognostic model for diffuse large B-cell lymphoma
Source: Front Oncol. 2023 Jan 12;12:1020566. doi: 10.3389/fonc.2022.1020566 (PMC9877310; doi:10.3389/fonc.2022.1020566)
Supplement: Supplementary Figure 1 — (A) Correlation analysis of CNV and survival in DLBCL. (B) The relationships between expression of CRGs and survival in DLBCL. (C) Correlation analysis of gene methylation of CRGs and survival in DLBCL. The bubble color from blue to red represents the hazard ratio from low to high, and bubble size is positively correlated with the Cox P value significance. The black outline border indicates Cox P value ≤ 0.05. [file DataSheet_1.zip › Supplementary Figure/Supplementary_Table1.pdf]

Supplementary Table 1 The clinical features of the samples in the experiments

ID	Gender	Age	COO	ECOG	Stage	LDH	Extranodal_sites
P1	Male	42	non-GCB	0	4	198	>=2
P2	Female	77	non-GCB	0	3	255	<2
P3	Male	46	non-GCB	0	NA	NA	NA
P4	Female	59	NA	0	4	379	<2
P5	Male	67	non-GCB	0	4	263	>=2
P6	Male	58	GCB	0	4	464	>=2
P7	Female	56	non-GCB	0	4	179	<2

Treatment	Gene
RCHOP	MYC(+), Bcl-2(-), Bcl-6(-)
RCHOP	NA
Untreated	NA
Untreated	MYC(+), Bcl-2(-), Bcl-6(-)
RCHOP	NA
RCHOP	MYC(+), Bcl-2(-), Bcl-6(-)
RCHOP	NA
