# Supplementary figures and images for: Development and validation of a cuproptosis-associated prognostic model for diffuse large B-cell lymphoma
Source: Front Oncol. 2023 Jan 12;12:1020566. doi: 10.3389/fonc.2022.1020566 (PMC9877310; doi:10.3389/fonc.2022.1020566)

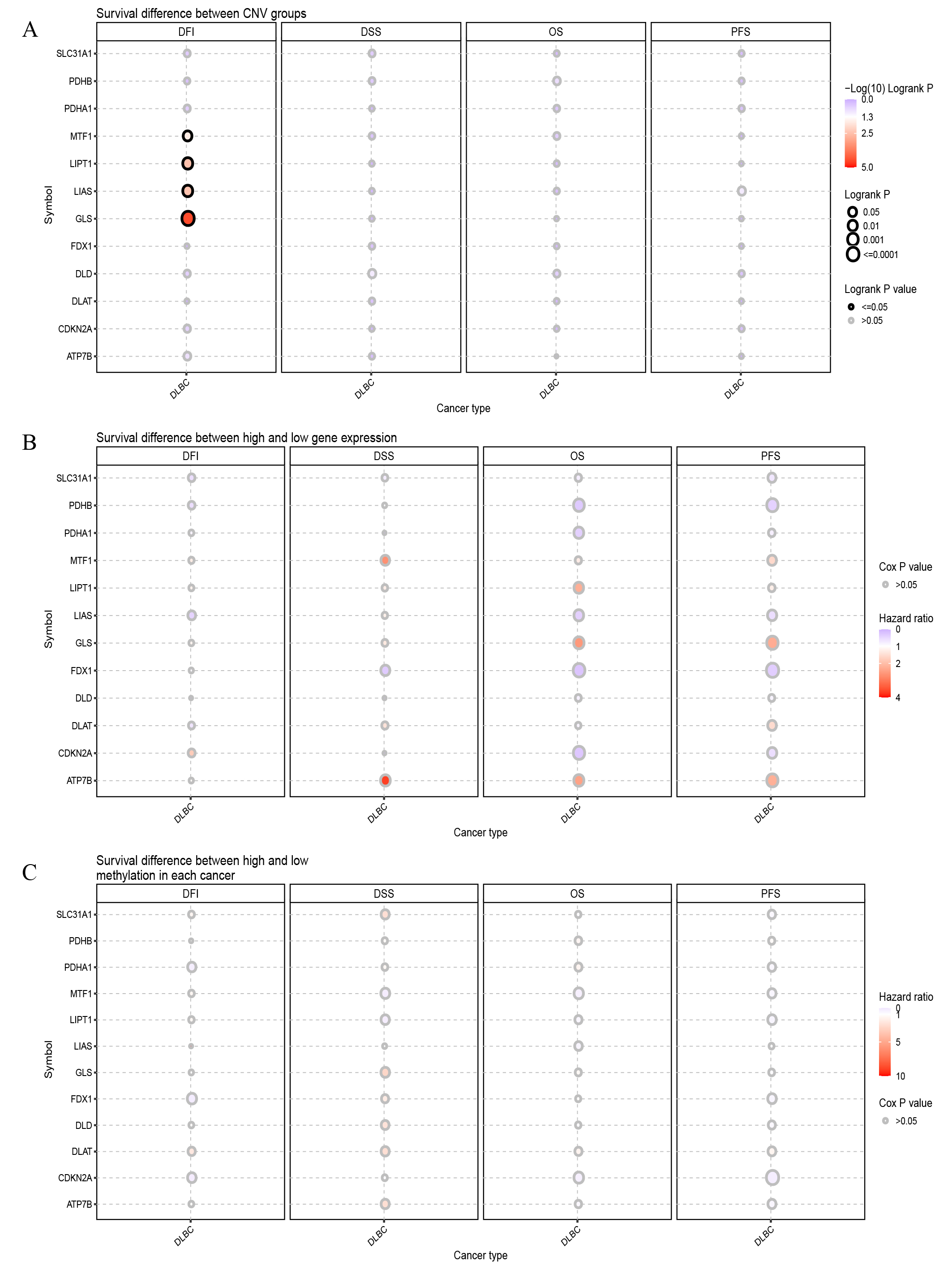

Supplement: Supplementary Figure 1 — (A) Correlation analysis of CNV and survival in DLBCL. (B) The relationships between expression of CRGs and survival in DLBCL. (C) Correlation analysis of gene methylation of CRGs and survival in DLBCL. The bubble color from blue to red represents the hazard ratio from low to high, and bubble size is positively correlated with the Cox P value significance. The black outline border indicates Cox P value ≤ 0.05. [file DataSheet_1.zip › Supplementary Figure/FigureS1_new.jpg]

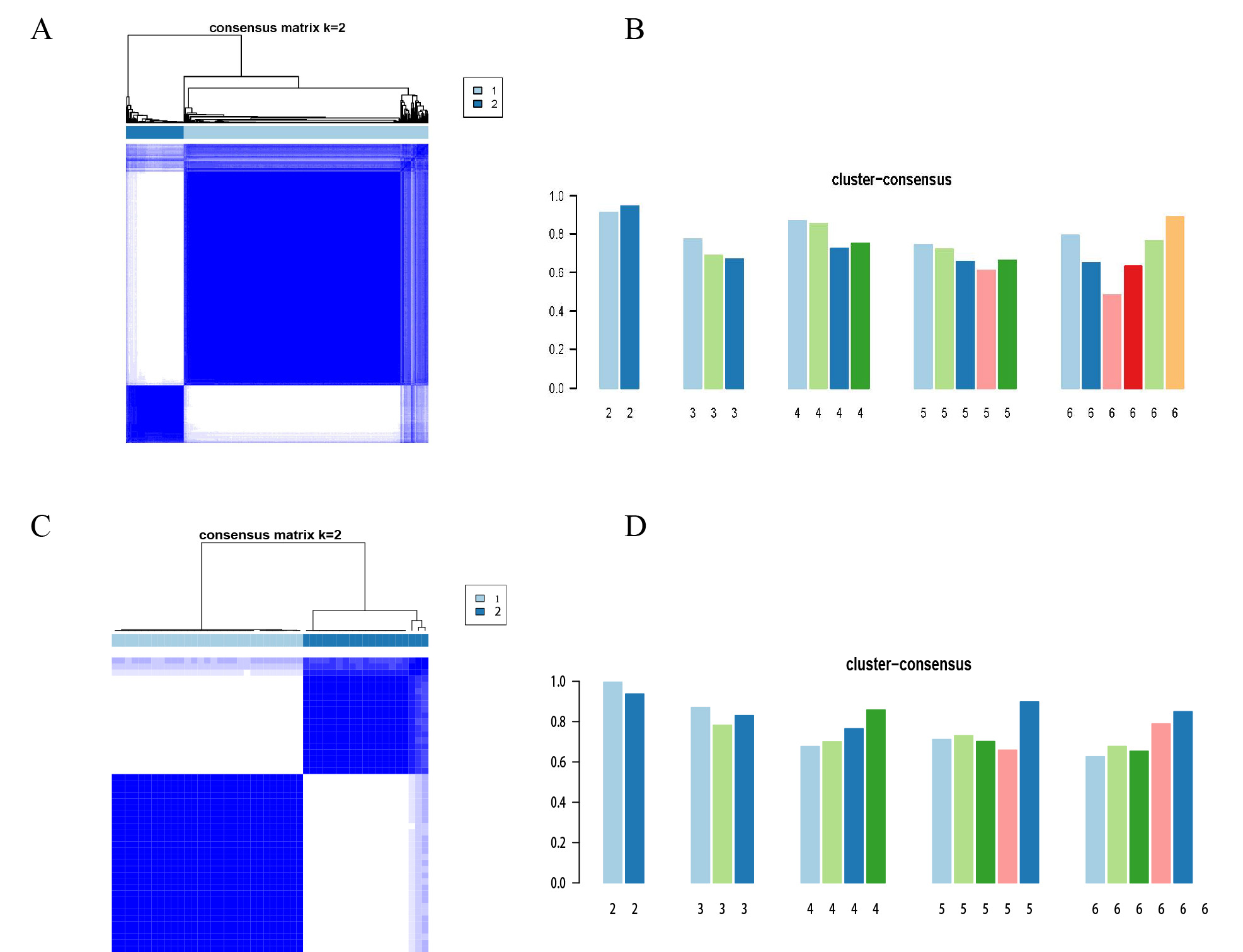

Supplement: Supplementary Figure 1 — (A) Correlation analysis of CNV and survival in DLBCL. (B) The relationships between expression of CRGs and survival in DLBCL. (C) Correlation analysis of gene methylation of CRGs and survival in DLBCL. The bubble color from blue to red represents the hazard ratio from low to high, and bubble size is positively correlated with the Cox P value significance. The black outline border indicates Cox P value ≤ 0.05. [file DataSheet_1.zip › Supplementary Figure/FigureS2_new.jpg]

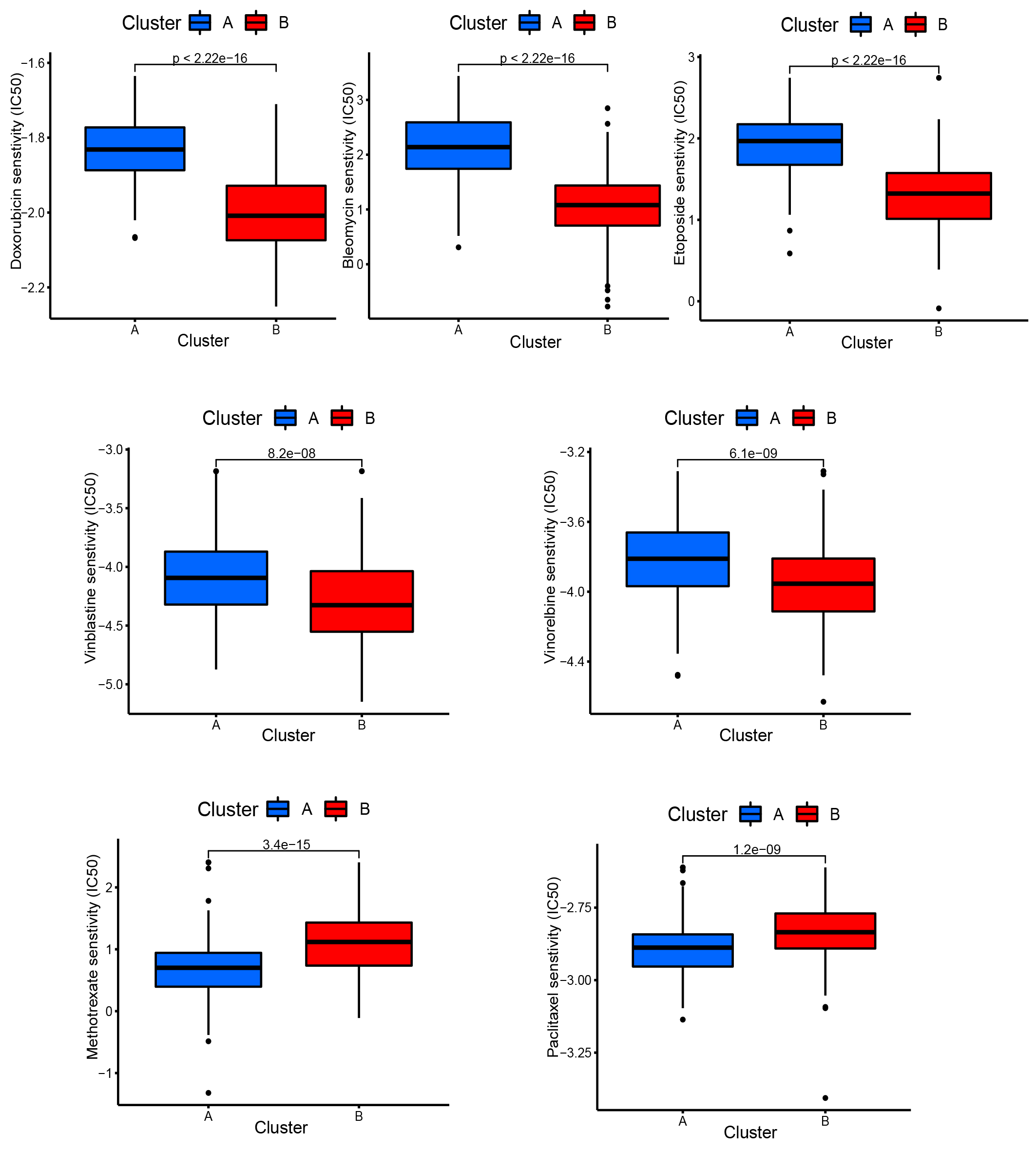

Supplement: Supplementary Figure 1 — (A) Correlation analysis of CNV and survival in DLBCL. (B) The relationships between expression of CRGs and survival in DLBCL. (C) Correlation analysis of gene methylation of CRGs and survival in DLBCL. The bubble color from blue to red represents the hazard ratio from low to high, and bubble size is positively correlated with the Cox P value significance. The black outline border indicates Cox P value ≤ 0.05. [file DataSheet_1.zip › Supplementary Figure/FigureS3_new.jpg]

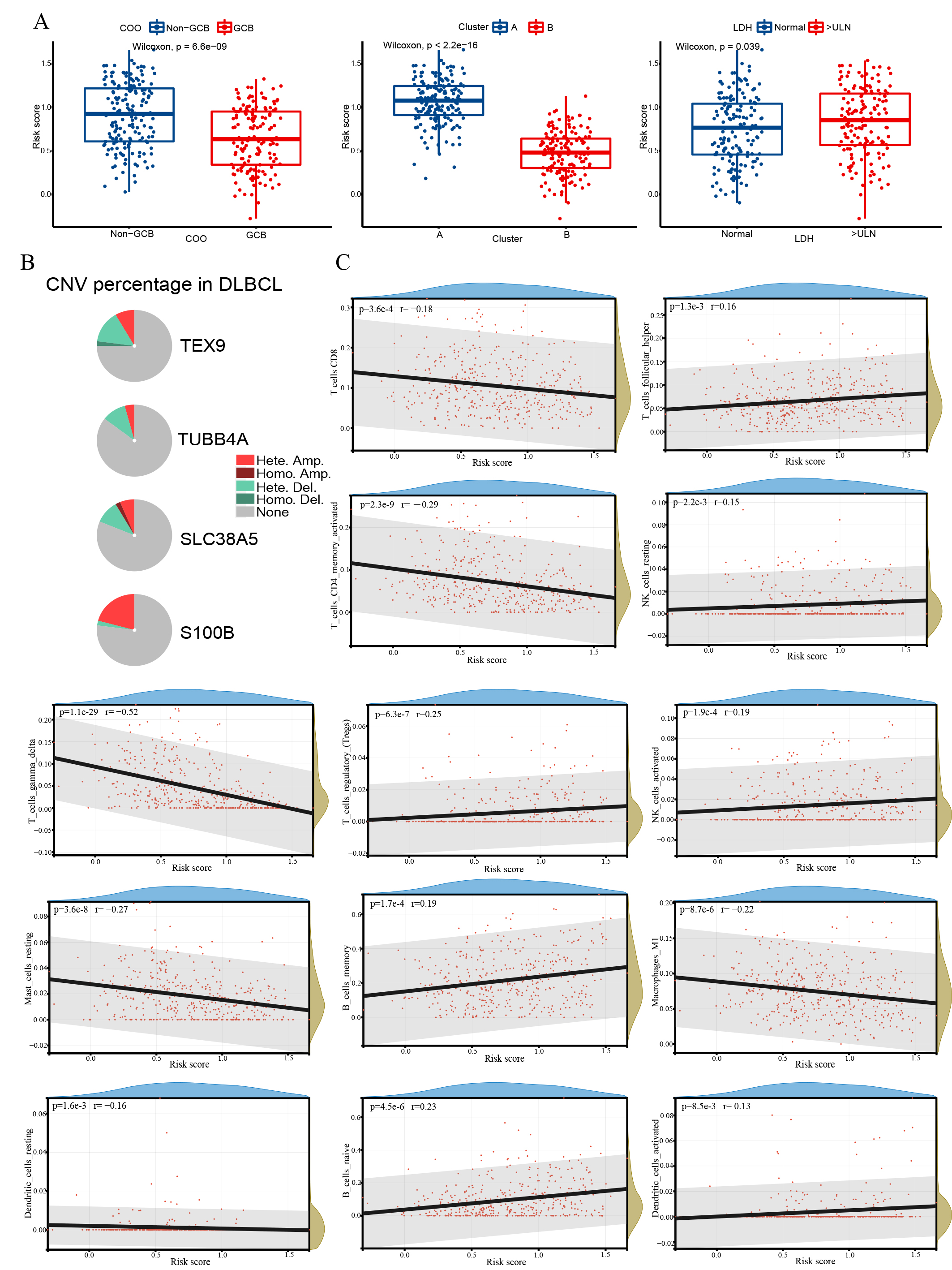

Supplement: Supplementary Figure 1 — (A) Correlation analysis of CNV and survival in DLBCL. (B) The relationships between expression of CRGs and survival in DLBCL. (C) Correlation analysis of gene methylation of CRGs and survival in DLBCL. The bubble color from blue to red represents the hazard ratio from low to high, and bubble size is positively correlated with the Cox P value significance. The black outline border indicates Cox P value ≤ 0.05. [file DataSheet_1.zip › Supplementary Figure/FigureS5_new.jpg]

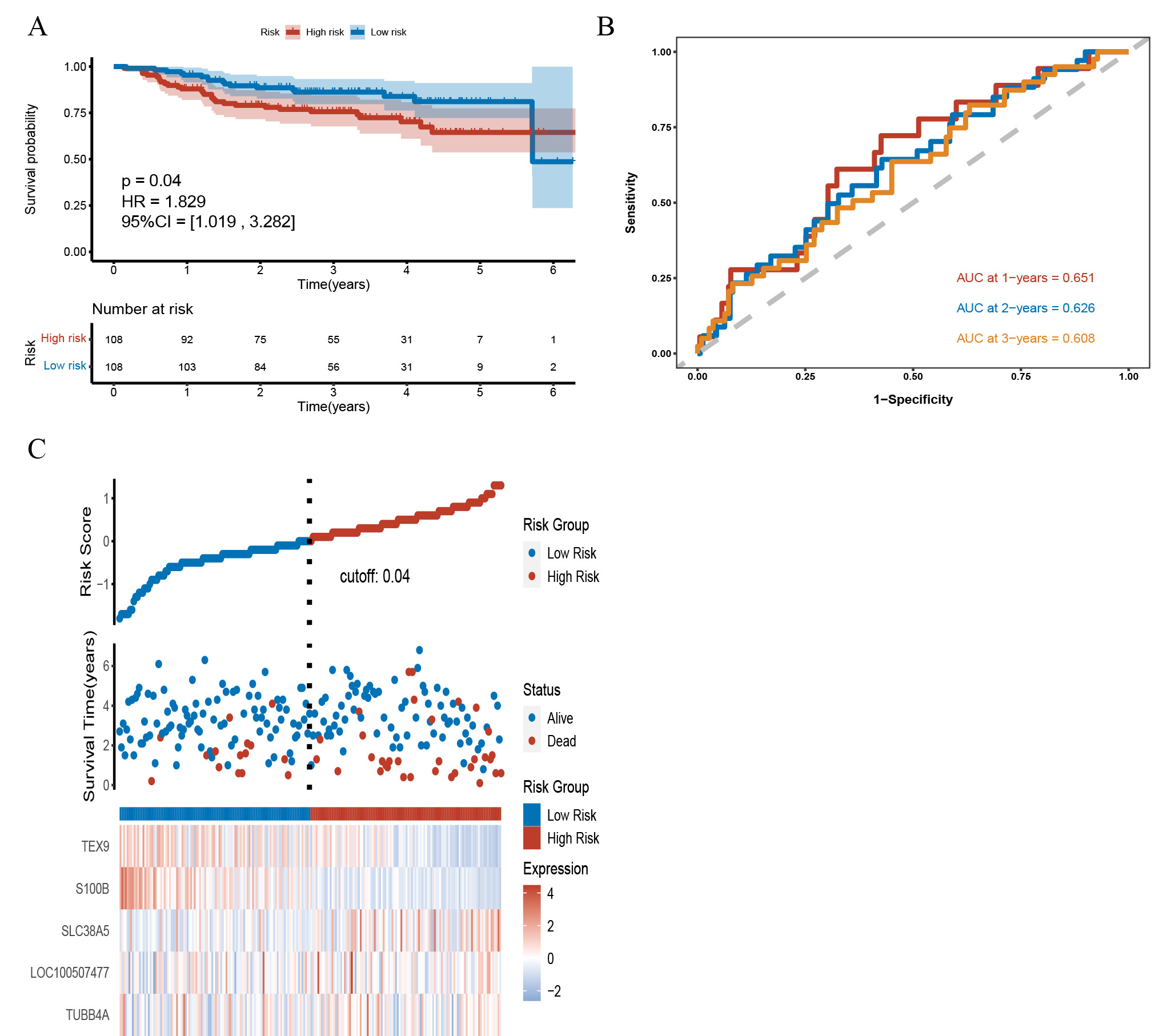

Supplement: Supplementary Figure 1 — (A) Correlation analysis of CNV and survival in DLBCL. (B) The relationships between expression of CRGs and survival in DLBCL. (C) Correlation analysis of gene methylation of CRGs and survival in DLBCL. The bubble color from blue to red represents the hazard ratio from low to high, and bubble size is positively correlated with the Cox P value significance. The black outline border indicates Cox P value ≤ 0.05. [file DataSheet_1.zip › Supplementary Figure/FigureS4_new4.jpg]
